# Characterization of argonaute nucleases from mesophilic bacteria *Pseudobutyrivibrio ruminis*

**DOI:** 10.1186/s40643-024-00797-x

**Published:** 2024-10-07

**Authors:** Xiaoyi Xu, Hao Yang, Huarong Dong, Xiao Li, Qian Liu, Yan Feng

**Affiliations:** State Key Laboratory of Microbial Metabolism, School of Life Sciences and Biotechnology, Shanghai Jiao Tong University, Shanghai, 200240 People’s Republic of China

**Keywords:** *Pseudobutyrivibrio ruminis* Argonaute, Mesophilic Argonaute, Prokaryotic Argonaute, Endonuclease, DNA cleavage

## Abstract

Mesophilic Argonautes (Agos) from microbial resources have received significant attention due to their potential applications in genome editing and molecular diagnostics. This study characterizes a novel Ago from *Pseudobutyrivibrio ruminis* (*Pr*Ago), which can cleave single-stranded DNA using guide DNA (gDNA). *Pr*Ago, functioning as a multi-turnover enzyme, effectively cleaves DNA using 5′-phosphate gDNA, 14–30 nucleotides in length, in the presence of both Mn^2+^ and Mg^2+^ ions. *Pr*Ago demonstrates DNA cleavage activity over a broad pH range (pH 4–12), with optimal activity at pH 11. As a mesophilic enzyme, *Pr*Ago cleaves efficiently DNA at temperatures ranging from 25 to 65 °C, particularly at 65 °C. *Pr*Ago does not show strong preferences for the 5′-nucleotide in gDNA. It shows high tolerance for single-base mismatches, except at positions 13 and 15 of gDNA. Continuous double-nucleotide mismatches at positions 10–16 of gDNA significantly reduce cleavage activity. Furthermore, *Pr*Ago mediates DNA-guided DNA cleavage of AT-rich double stranded DNA at 65 °C. Additionally, molecular dynamic simulations suggest that interactions between the PAZ domain and different nucleic acids strongly influence cleavage efficiency. These findings expand our understanding of Protokaryotic Agos and their potential applications in biotechnology.

**Graphical Abstract:**

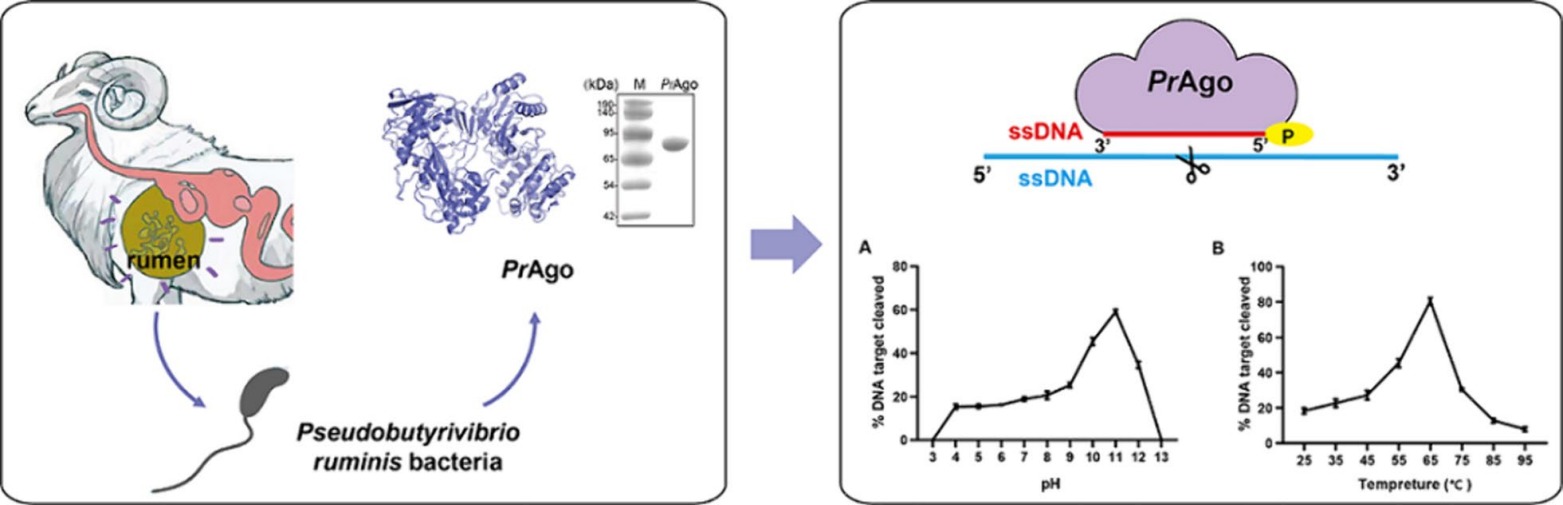

**Supplementary Information:**

The online version contains supplementary material available at 10.1186/s40643-024-00797-x.

## Introduction

The Ago protein family is present in archaea, prokaryotes, and eukaryotes (Willkomm et al. 2015; Bobadilla Ugarte et al. 2023; Swarts et al. 2014). Ago proteins are widely recognized for their role as nucleases in the small RNA-mediated gene silencing network in eukaryotes (Lisitskaya et al. 2018; Wang et al. 2009). Prokaryotic Argonauts (pAgos) and eukaryotes have discernible preferences, with pAgos capable of cleaving RNA/DNA targets using RNA/DNA as guides (Lisitskaya et al. 2018; Jiang et al. 2022; Koopal et al. 2022; Olovnikov et al. 2013). CRISPR and pAgo are nucleic acid-guided defense systems that protect prokaryotes against the invasion of mobile genetic elements. Given the impact of CRISPR on genome editing, there is curiosity about the gene-editing potential of PAM-free pAgo (Hegge et al. 2018; Shen et al. 2023; Esyunina et al. 2023). pAgos derived from thermophilic bacteria, with their optimal activity temperature exceed 65 °C, exhibit low endonuclease activity at 20–37 °C, rendering them unsuitable for genome editing (Hegge et al. 2019; Swarts et al. 2015; Fang et al. 2022). The discovery of mesophilic Agos will establish the foundation for prokaryotic Ago-based applications in biotechnology.

Recently, significant advancements have been made in the study of mesophilic Ago (Li et al. 2022a; Cao et al. 2019; Sun et al. 2022a; Wang et al. 2024; Zhao et al. 2024; Ye et al. 2022; Graver et al. 2024). *Natronobacterium gregory* Ago (*Ng*Ago) is a nucleic acid endonuclease with ssRNA or ssDNA as a guide (Dong et al. 2023; Qi et al. 2016; Lee et al. 2021). *Ng*Ago has demonstrated the ability to use RNA as a guide to modulate gene expression in mammalian cells or selectively bind to specific genomic DNA loci to disrupt gene transcription in zebrafish (Dong et al. 2023; Qi et al. 2016). Furthermore, both *Ng*Ago and *Clostridium butyricum* Ago (*Cb*Ago) have been observed to enhance homology recombination in bacteria (Fu et al. 2019; Huang et al. 2023; Lee et al. 2021; Esyunina et al. 2023). However, mesophilic Agos lack the capacity to unwind double-stranded DNA (dsDNA), unlike CRISPR, and are currently unable to perform precise genome cleavage as effectively as CRISPR (Jiang et al. 2022; Esyunina et al. 2023; Hegge et al. 2018; Kaya et al. 2016; Lee et al. 2021). In the field of virus detection, mesophilic pAgo has also demonstrated significant application potential. For instance, the *Km*Ago can be combined with reverse transcriptase at moderate temperatures for detecting SARS-CoV-2 (Li et al. 2022b). Additionally, based on *Cb*Ago, a fluorescence biosensor was developed for detecting viable *Salmonella typhimurium* and for nucleic acid quantification at ambient temperature (Zhao et al. 2024; Wang et al. 2024). Further exploration is essential to uncover a broader spectrum of stable mesophilic Ago and their practical applications.

In this study, we characterized a mesophilic *Pr*Ago from *Pseudobutyrivibrio ruminis*, which can use ssDNA as guides to specifically cleave ssDNA targets under wide pH conditions and temperature ranges. It also shows programmable cleavage of dsDNA at 65 °C. In addition, molecular dynamic (MD) simulations suggest that interactions between the PAZ domain and various dsDNAs influence cleavage efficiency. These findings have broadened our understanding of pAgo proteins and have the potential to advance the development of pAgo-based applications at moderate temperatures.

## Materials and methods

### Phylogenetic tree and sequence alignment

Sequence alignment was obtained when BLAST was used to search for *Cb*Ago sequence (WP_045143632.1) in the NCBI database. Analyses were performed using MEGA X. The evolutionary history was inferred using the Neighbor-Joining method, and multiple sequence alignment analyses were performed using ClustalW.

### Prediction of *Pr*Ago’s three-dimensional structure using SWISS-MODEL

We used SWISS-MODEL (expasy.org) to predict *Pr*Ago’s three-dimensional structure, with *Cb*Ago (PDB: 6QZK) as the template. The positions of metal ions were predicted using the Mg^2+^ coordinates of *Thermus thermophilus* Ago (*Tt*Ago) (PDB: 4NCB).

### Strains, plasmids and medium

*Escherichia coli* BL21 (DE3) Chemically Competent Cells (CD601-02) and Trans5α Chemically Competent Cells (CD201-01) were purchased from TransGen Biotech (Beijing, China). The recombinant plasmid pET-28a (+)-*Pr*Ago/*Cb*Ago, containing the synthesized codon-optimized Ago gene, was constructed by GenScript (China). Luria–Bertani (LB) medium (containing 10 g/L tryptone, 5 g/L yeast extract, and 10 g/L NaCl) was used for Ago expression.

### Protein expression and purification

The expression vector pET-28a (+)-*Pr*Ago/*Cb*Ago was transformed into the *E*. coli BL21 (DE3) strain. Positive clones were cultured in a shaker incubator at 37 °C and 180 rpm in LB medium containing 50 μg/mL kanamycin. After 12 h, 10 mL of bacterial culture was added to 1 L of LB medium containing 50 μg/mL kanamycin at 37 °C and 180 rpm until the OD_600_ value reached approximately 0.8. After cooling the medium on ice, Ago expression was induced with a final concentration of 0.5 mM isopropyl-β-d-1-thiogalactopyranoside (IPTG) at 18 °C for 18–20 h. The bacteria were harvested by centrifugation and resuspended in lysis buffer (20 mM Tris–HCl, 250 mM NaCl, 10 mM imidazole, 2% [v/v] glycerol, 0.05% [v/v] Triton X-100, pH 7.5–8.0). The bacteria were disrupted using a high-pressure homogenizer (Gefran, Italy) at 700–800 bar for 10 min. The lysate was then centrifuged for 30 min at 4 °C and 12,000 rpm, and the supernatants were purified using a nickel-nitrilotriacetic acid (Ni–NTA) affinity column. The Ni–NTA affinity column was eluted with elution buffer (20 mM Tris–HCl, 250 mM NaCl, 250 mM imidazole, 5 mM thioglycol, pH 7.5–8.0). The protein was applied to a heparin column (HiTrap Heparin HP, cytiva), eluted with a linear gradient of 125 mM–1 M NaCl. Gel Filtration separates impurities from protein samples. The purified protein was stored at − 80 °C.

### Single-stranded nucleic acid cleavage activity assays

The 5′-phosphorylated (P)/hydroxylated (OH) ssDNA guides (gDNA) and ssDNA targets were commercially synthesized by Sangon (Shanghai, China) (Table S1). The 5′-P/OH ssRNA guides (gRNA) and ssRNA targets were commercially synthesized by GenScript (Shanghai, China). Most cleavage assays were conducted using 0.5 μM *Pr*Ago, 0.5 μM ssDNA or ssRNA guides, and 0.1 μM ssDNA or ssRNA targets in a reaction buffer (20 mM Tris–HCl, 10 mM NaCl, 3 mM MnCl_2_, 0.1 mM DDT, pH 7.5). *Pr*Ago and the guide were incubated for 15 min at room temperature. The reaction system was then placed on ice, Target DNA was added, mixed, centrifuged, and immediately incubated in PCR machine. Different reaction systems will be indicated in the diagram. The reaction was halted using 2X miRNA Deionized Formamide Gel Loading Buffer (B548651-0001, Sangon, China). Samples were separated on 16% denaturing polyacrylamide gels, stained with GelRed (Biotium, Fremont, USA) for 20 min, and visualized using a Gel Image System (Tanon-3500BR, Tanon, China).

### Effect of pH, temperature, divalent metal ions on *Pr*Ago activity

To investigate whether *Pr*Ago can cleave ssDNA under different pH conditions, various buffers were used: glycine–HCl (pH 3.0 to 5.0), Tris–HCl (pH 6.0 to 8.0), glycine–NaOH (pH 9.0 to 10.0), and KCl–NaOH (pH 11.0 to 13.0) (Fu et al. 2014; Terada et al. 1999; Chen et al. 2019; Liang et al. 2014; Maalej et al. 2014; Benchouaia et al. 2024). Reaction systems will be indicated in the diagram. To investigate the effect of temperature on the reaction, we first added target DNA on ice, then mixed and centrifuged the samples. Immediately after centrifugation, the samples were placed into PCR instruments pre-set to different constant temperatures for the reaction. To investigate the effect of divalent metal ions on the reaction, 0.5 mM of various divalent metal ions (CaCl_2_, CoCl_2_, CuCl_2_, MgCl_2_, MnCl_2_, NiCl_2_, and ZnCl_2_) were added to the reaction system. To explore the optimal manganese ion concentration, different final Mn^2+^ concentrations (0, 5, 10, 20, 50, 100, 200, 300 and 500 mM) were added to the reaction buffer, reaction systems will be indicated in the diagram.

### Effect of the length of 5′-P gDNA on *Pr*Ago activity

Different lengths of 5′-P gDNA targets and ssDNA targets were synthesized and kept the reaction buffer unchanged. Data was processed using ImageJ and GraphPad Prism 7.0.

### Effect of guide/target mismatch on *Pr*Ago activity

The 5′-P gDNAs with single-nucleotide mismatches or double-nucleotide mismatches at different positions were synthesized separately at Sangon (Shanghai, China). To investigate the effect of the first nucleotides of the target DNA on *Pr*Ago activity, 18 nt 5′-P gDNAs (G1A, G1T, G1G, G1C) were synthesized. Data was processed using ImageJ and GraphPad Prism 7.0.

### Double-stranded nucleic acids cleavage activity assays

The reaction buffer was described previously, but the reaction time was extended to 3 h, and the reaction temperature was shown in figure. Additionally, 700 ng of plasmid pUC19 was added to each reaction system, the other reaction components are indicated in the diagram. Each pair of 5′-P gDNAs is complementary and specifically targets different GC contents of pUC19. To stop the reaction, proteinase K and CaCl_2_ were used to reaction system for 2 h. Experimental results were detected using 1% agarose gel electrophoresis.

### Molecular dynamics analysis of *Pr*Ago

We use HDOCK server for protein-gDNA docking. The HDOCK web server is available at http://hdock.phys.hust.edu.cn/. gDNA is modeled by Amber software. Gromacs2022.3 software was used for molecular dynamics (MD) simulation. Simulation conditions were performed at a static temperature of 300 K and atmospheric pressure (1 Bar). Amber99sb-Idn was used as the force field. The solvent was a water molecule (Tip3p water model) and the total charge of the simulation system was neutralized by adding an appropriate amount of Na^+^ ions. The simulation system adopts the steepest descent method to minimize energy, and then carries out the isothermal isovolumic ensemble (NVT) equilibrium and isothermal isovolumic ensemble (NPT) equilibrium at 500,000 steps, respectively, with the coupling constant of 0.1 ps and the duration of 500 ps. Finally, the free MD simulation was performed. The process was 5,000,000 steps, the step length was 2 fs, and the total duration was 100 ns. After the simulation, the software’s built-in tool was used to analyze the trajectory, calculate the root mean square variance (RMSD), root mean square fluctuation (RMSF) and other data.

### Fluorescence polarization assays

Fluorescence polarization assays were conducted in black 96-well microplates, with each well (100 μL) containing 5 nM of 3′-6-FAM-labeled gDNA and varying concentrations (0–1 μM) of *Pr*Ago in reaction buffer, incubated at 37 °C for 20 min. Polarization was measured using a plate reader (Spark, Tecan) with excitation at 498 nm and emission at 520 nm. The experiments were performed in triplicate. Data were analyzed using GraphPad Prism 7.0 for nonlinear regression curve fitting to calculate the K*D* value.

## Results

### *Pr*Ago can use 5′-P DNA guides to cleave ssDNA targets

**Fig. 1:**
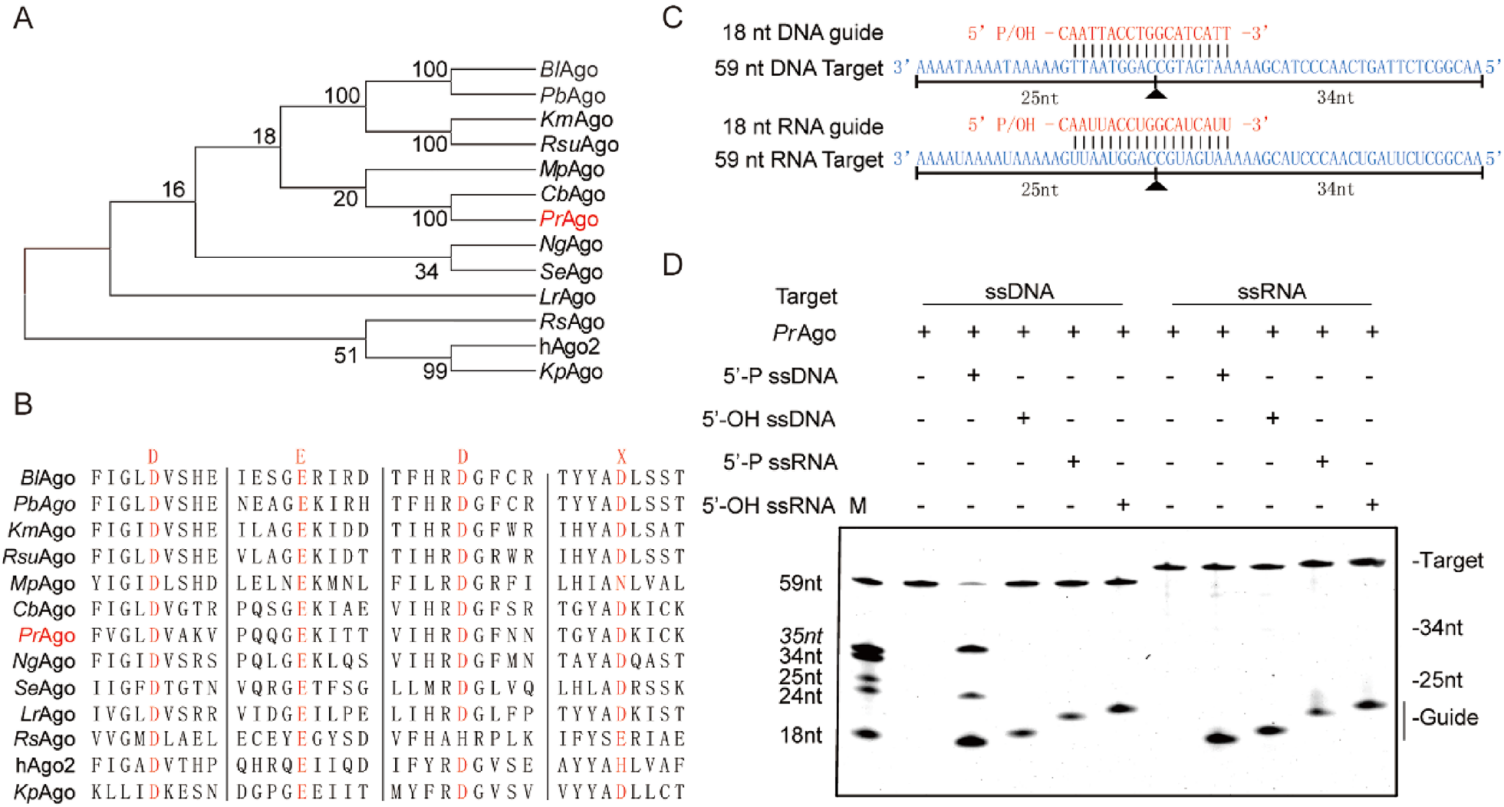
Phylogenetic analysis and cleavage activity assay of *Pr*Ago. **A** Phylogenetic analysis of *Pr*Ago based on amino acid sequence. The evolutionary history was inferred using the Neighbor-Joining method. *Bl*Ago: *Brevibacillus laterosporus* Ago; *Pb*Ago: *Paenibacillus borealis* Ago; *Km*Ago: *Kurthia massiliensis* Ago; *Rsu*Ago: *Rummeliibacillus suwonensis* Ago; *Mp*Ago: *Marinitoga piezophila* Ago; *Cb*Ago: *Clostridium butyricum* Ago; *Pr*Ago: *Pseudobutyrivibrio ruminis* Ago; *Ng*Ago: *Natronobacterium gregory* Ago; *Se*Ago: *Synechococcus elongatus* Ago; *Rs*Ago: *Rhodobacter Sphaeroides* Ago; hAgo2: *Homo sapiens* Ago; *Lr*Ago: *Limnothrix rosea* Ago; *Kp*Ago: *Kluyveromyces polysporus* Ago. **B** Multiple sequence alignment of *Pr*Ago with several other mesophilic Agos. Red mark means the key catalytic residues. **C** Synthetic DNA/RNA guides (red) and DNA/RNA targets (blue). The cleavage positions are indicated with a black triangle, black lines indicate the predicted 25- and 34-nt cleavage products. **D** Cleavage activity assays of *Pr*Ago were performed ssDNA or ssRNA target in reaction buffer for 30 min at 37 °C

In our quest to explore and discover mesophilic Agos that remain stable in extreme environments, we identified *Pr*Ago in the rumen of cattle and sheep, originating from *Pseudobutyrivibrio ruminis* bacteria (Pidcock et al. 2021; Kasperowicz et al. 2012). We used the BLASTp program’s web interface to search for mesophilic pAgos and searched in the NCBI database using the BLASTp program. Subsequently, we constructed a phylogenetic tree of mesophilic Ago proteins from various strains (Fig. 1A). Multiple sequence alignment revealed that *Pr*Ago contains the conserved DEDD catalytic residues necessary for its cleavage activity, suggesting that *Pr*Ago may be catalytically active (Fig. 1B). The recombinant *Pr*Ago protein was successfully expressed in *E. coli* BL21 (DE3) using a codon-optimized gene and pET28a expression system, and it was subsequently purified (Figure S1A). To determine if there are copurified nucleic acids, we measured the A260/A280 ratio of the purified *Pr*Ago. The sample from the nickel column purification displayed an A260/280 ratio of 0.83; after heparin column affinity, the ratio decreased to 0.56. To further validate the absence of nucleic acids in the purified *Pr*Ago, we employed proteinase K to digest proteins in the samples. No copurified nucleic acids were detected after heparin column affinity (Figure S1B). Both the A260/A280 ratio and the results of nucleic acids isolation indicated that the purified *Pr*Ago was free of nucleic acids (Liu et al. 2021b; Hegge et al. 2019). We utilized SWISS-MODEL to predict three-dimensional structure of *Pr*Ago, using *Cb*Ago (NDB: 6QZK) as the prediction template (Figure S2). Similar to other classic long Agos, *Pr*Agos was predicted to have a bilobular architecture, with the PAZ lobe (N, L1 and PAZ domains) connected by L2 to the PIWI lobe (MID and PIWI domains). To confirm whether *Pr*Ago is indeed an active nuclease, we performed in vitro activity assays in which *Pr*Ago was loaded with either synthetic ssDNA or ssRNA (18 nt) (Fig. 1C). Next the complexes were incubated at 37 °C with 59 nt complementary ssRNA or ssDNA as target. It revealed that *Pr*Ago was a DNAase and preferred ssDNAs as the guide (Fig. 1D). Since *Cb*Ago is known for its high enzymatic cleavage rate and shares high similarity with *Pr*Ago, we compared the cleavage activity of *Pr*Ago with that of *Cb*Ago (Vaiskunaite et al. 2022). The products were measured using real-time fluorescent quantification. The results revealed that *Pr*Ago exhibits slightly higher cleavage efficiency than *Cb*Ago under evaluated conditions (Figure S3). The majority of characterized pAgos require pre-incubation with guides before targeting cleavage in vitro (Kropocheva et al. 2021; Jiang et al. 2022; Liu et al. 2021b; Ye et al. 2022). We then measured the effect of various pre-incubation times. Our observations suggest that pre-incubation of *Pr*Ago and gDNAs leads to higher cleavage efficiency compared to non-incubated conditions. However, the duration of pre-incubation may have little impact on cleavage efficiency (Figure S4).

### The activity of *Pr*Ago was affected by pH, divalent metal ions and temperature

**Fig. 2:**
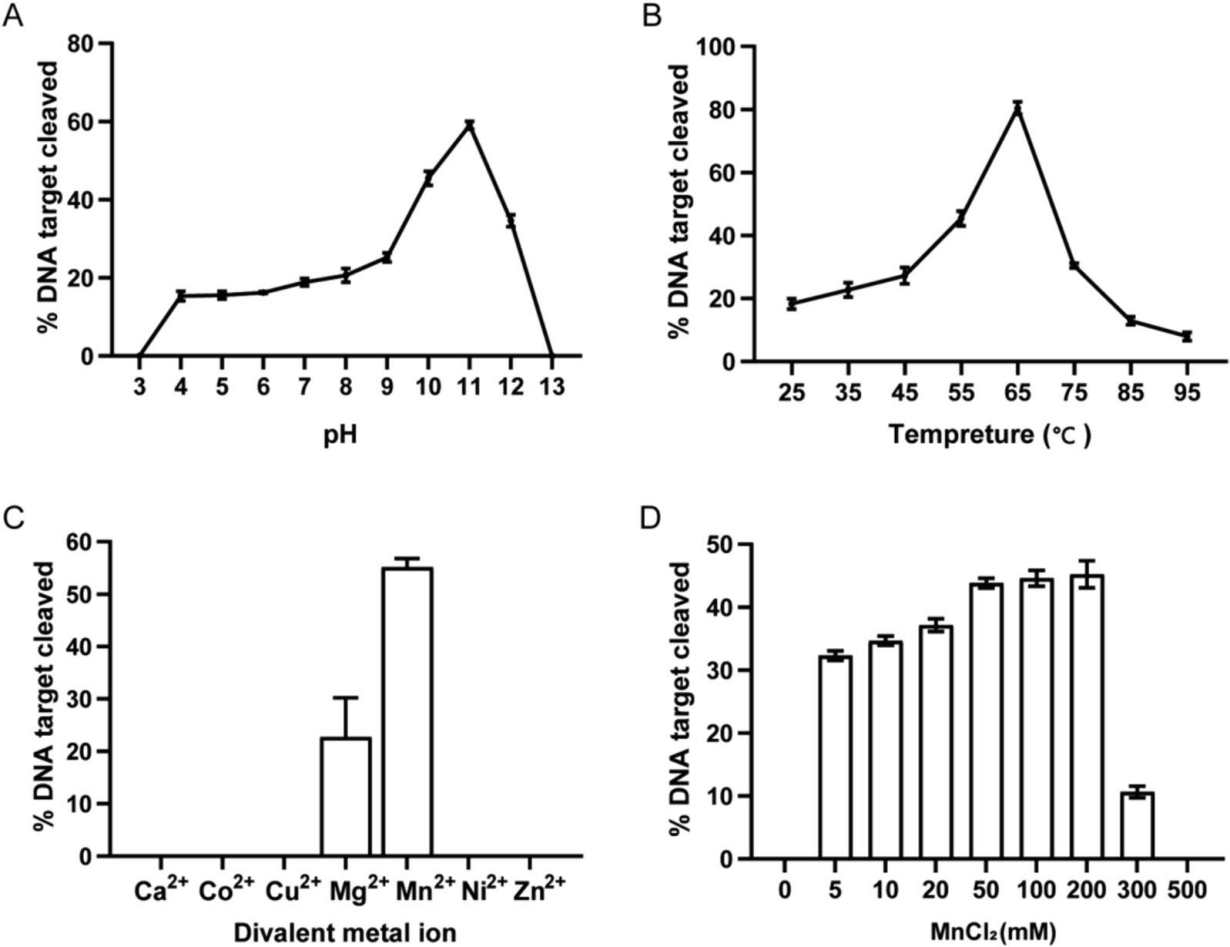
Effects of pH, temperature and metal ion on *Pr*Ago cleavage activity. **A** Effect of pH on *Pr*Ago activity mediated by the 5′-P gDNA at 37 °C. Reaction buffer contain 25 mM different pH buffer, 25 mM KCl, 3 mM MnCl_2_, 0.5 M NaCl, 0.5 μM *Pr*Ago, 0.5 μM gDNA, and 2 μM 5′FAM-target DNA. The x-axis represents the pH of the glycine buffer. The error bars above represent the SDs of three independent experiments. **B** Effect of temperature on *Pr*Ago activity. Reaction buffer contains 20 mM Tris 8.0, 1 mM NaCl, 3 mM MnCl_2_, 10 mM DTT, 0.5 μM *Pr*Ago, 0.5 μM gDNA, and 2 μM 5′FAM-target DNA. The error bars above represent the SDs of three independent experiments. **C** *Pr*Ago displays Mn^2+^ and Mg^2+^ mediated ssDNA target cleavage. The error bars above represent the SDs of three independent experiments. **D** Effect of MnCl_2_ concentrations on *Pr*Ago enzyme activity. Reaction buffer contains 20 mM Tris 8.0, 1 mM NaCl, 10 mM DTT, 0.5 μM *Pr*Ago, 0.5 μM gDNA, 2 μM 5′FAM-target DNA and different concentrations of MnCl_2_. The error bars above represent the SDs of three independent experiments

To further determine the prerequisites for *Pr*Ago-mediated target cleavage, we tested the influence of pH, temperature, divalent metal ions type and salt concentration on ssDNA cleavage. *Pseudobutyrivibrio ruminis* bacteria were isolated and identified from the rumen of animals (Kasperowicz et al. 2012; Pidcock et al. 2021). Therefore, we want to observe the activity of *Pr*Ago in buffers with different pH. We detected its catalytic activities at pH ranging from 3 to 13 using 40 nt ssDNA and 18 nt ssDNA guide at 37 °C, and we found that *Pr*Ago exhibited cleavage ssDNA at a wide pH range from 4 to 12, especially at pH 11 (Fig. 2A). To determine the temperature range for *Pr*Ago, we tested its cleavage activity at temperatures ranging from 25 to 95 °C (Garcia-Quintans et al. 2019; Dong et al. 2021). The results indicated that *Pr*Ago was most active at temperatures 65 °C (Fig. 2B). To determine whether the cleavage products observed at 75 °C, 85 °C, and 95 °C were due to decreased enzyme activity at high temperatures, we conducted experiments with varying time gradients at these temperatures. The results showed that cleavage products appeared after 15 min and did not increase with time (Figure S5). Therefore, we conclude that *Pr*Ago is not a thermophilic enzyme. Cleavage by *Pr*Ago with the 5′-P DNA guide was only observed when Mn^2+^ or Mg^2+^ ions were provided (Fig. 2C), and *Pr*Ago exhibited high cleavage efficiency when Mn^2+^ concentrations ranged from 5 to 200 mM (Fig. 2D). We conducted a time gradient experiment under conditions of 300 mM MnCl_2_. Reaction products appeared after 15 min but did not increase with time. We conclude that *Pr*Ago cannot tolerate 300 mM MnCl_2_ (Figure S6). Na^+^ plays an essential role in maintaining the stability of pAgos, and *Pr*Ago can cleave ssDNA when Na^+^ concentrations ranged from 1 to 2000 mM (Figure S7A). The time gradient experiment indicated that under reaction conditions of 1 M or 2 M NaCl, the reaction products increased with time although with a decreased rate. Therefore, we conclude that *Pr*Ago can tolerate high concentrations of Na^+^ (Figure S7B).

To further investigate the catalytic properties of *Pr*Ago, we monitor cleavage efficiency under conditions of variable ratios of [*Pr*Ago-gDNA]: [Target] ([Target] >> [*Pr*Ago: gDNA] or [Target] >> [*Pr*Ago: gDNA]) (Liu et al. 2023; Li et al. 2022a; Kuzmenko et al. 2019; Wang et al. 2009). The results showed that when *Pr*Ago and gDNA were significantly lower than target DNA (at a ratio of 0.25:1), target DNA was cleaved about 75%. When *Pr*Ago and gDNA were significantly greater than or similar to target DNA, the amount of cleaved target DNA was approximately 95% (Figure S8A, B). Therefore, we concluded that *Pr*Ago was a multiple-turnover enzyme at 65 °C. For the reason that target DNA was not fully cleaved when [*Pr*Ago-gDNA]: [Target] at ratios of 0.25 and 0.5, we speculated that *Pr*Ago may have gradually become inactive under prolonged conditions at 65 °C or the reaction time needed to be extended.

### *Pr*Ago mediates double-stranded DNA cleavage

**Fig. 3:**
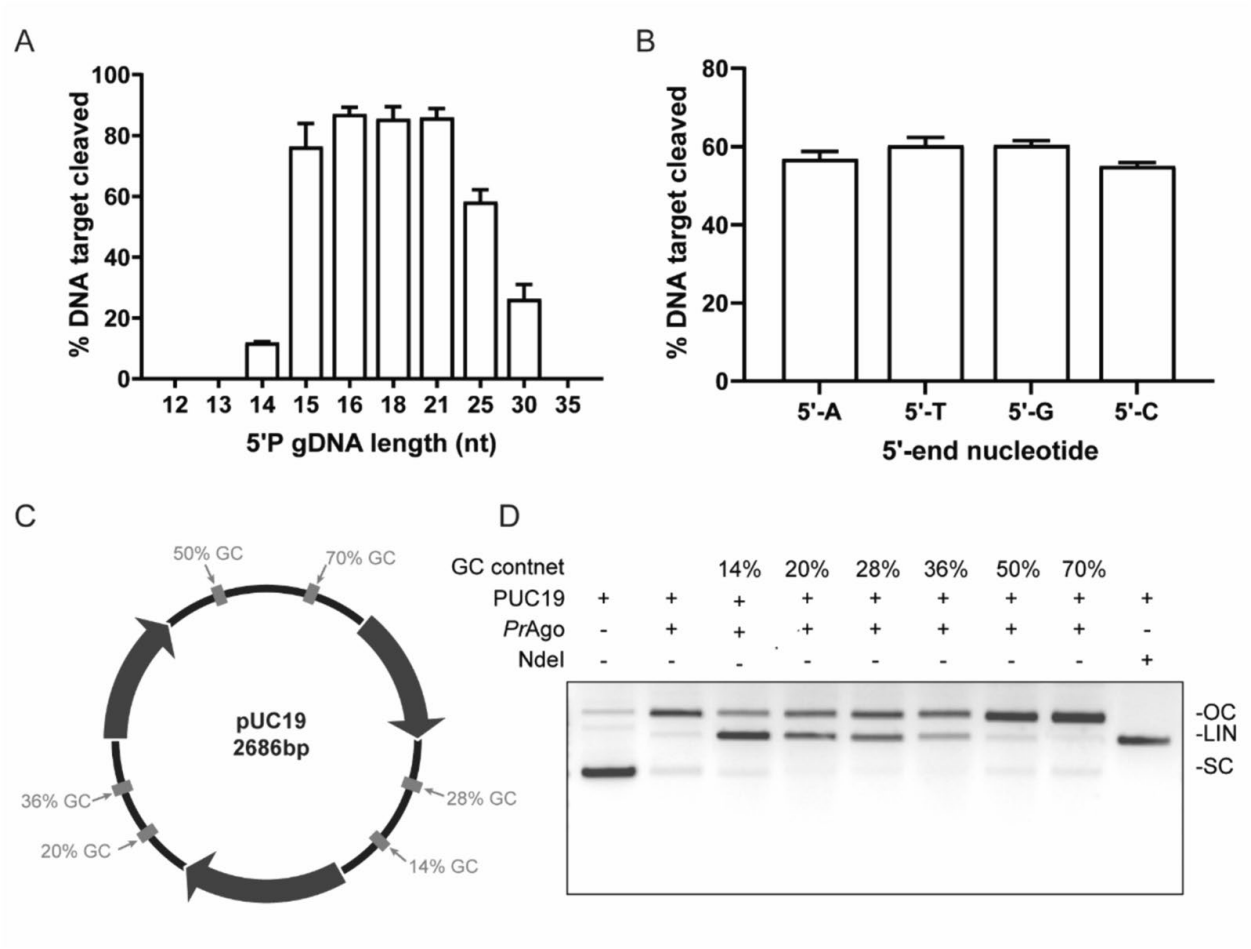
Cleavage of pUC19 by *Pr*Ago. **A** Dependence of DNA cleavage efficiency on gDNA length. The reaction was performed at 37 °C. The error bars above represent the SDs of three independent experiments. **B** Effect of the 5′-terminal nucleotide of the 5′-P gDNA on *Pr*Ago at 37 °C. The error bars above represent the SDs of three independent experiments. **C** Schematic overview of pUC19 target plasmid. Target sites are shown in grey. Percentages indicate the GC content of the 50 bp segments in which these target sites are located. **D** Plasmid cleavage in different target regions at 65 °C. Reaction buffer contains 20 mM Tris 8.0, 1 mM NaCl, 3 mM MnCl_2_, 10 mM DTT, 0.5 μM *Pr*Ago, 0.5 μM gDNA, 700 ng pUC19. *OC* open circular plasmid, *LIN* linearized plasmid, *SC* supercoiled plasmid

The length of the guide has been reported to affect the cleavage efficiency of pAgos (Kropocheva et al. 2021). We observed a highly efficient cleavage effect when the length of the gDNA within the range of 16–21 nt (Fig. 3A). To investigate the effect of the 5′-end nucleotides of guides on target cleavage of *Pr*Ago, various DNA guides were designed with different 5′-end nucleotides (Sun et al. 2022b). *Pr*Ago showed no significant preference for the 5′-end nucleotides of gDNA (Fig. 3B). Most pAgos can cleave low GC content regions of plasmids (Kuzmenko et al. 2019; Hegge et al. 2019; Liu et al. 2021b; Cao et al. 2019). To determine whether *Pr*Ago can cleave dsDNA substrates, we incubated *Pr*Ago and paired gDNA complexes with the target plasmid (pUC19) at 65 °C, and six dsDNA fragments with different GC content of 50 bp on plasmid pUC19 were used as targets (Fig. 3C). *Pr*Ago could cleave dsDNA fragments with a GC content of 28% or less (Fig. 3D). However, *Pr*Ago could not cleave the plasmid at 37 °C (Figure S9).

### Effect of guide-target mismatches on cleavage activity of *Pr*Ago

**Fig. 4:**
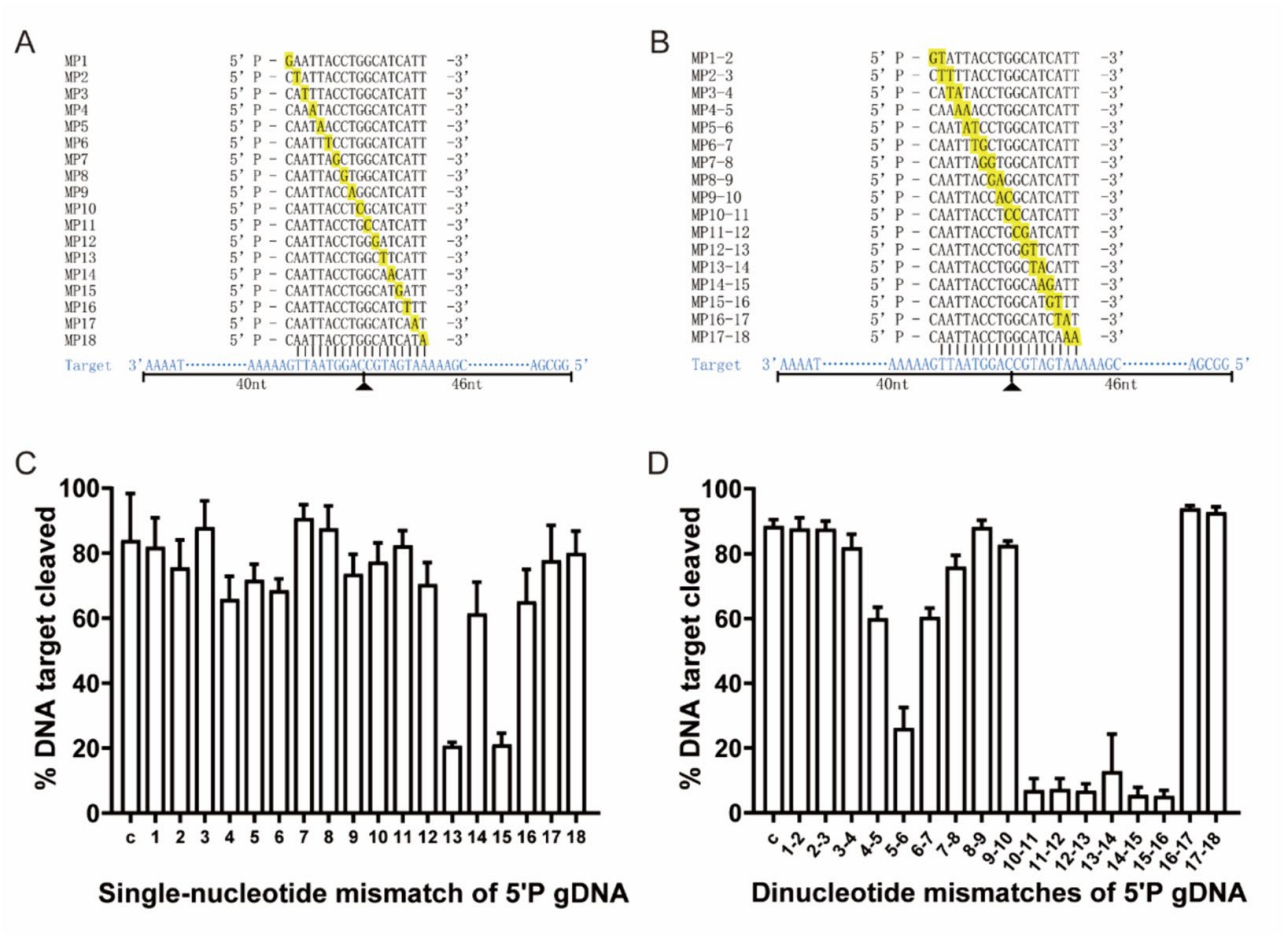
Effects of mismatch in guide-target duplex. **A** Schematic of gDNAs with single-base mismatch positions. **B** Schematic of gDNAs with double-base mismatch position. **C** Target ssDNA cleavage activity of *Pr*Ago loaded with single-nucleotide mismatched gDNA. The reaction was performed at 37 °C. The error bars above represent the SDs of three independent experiments. **D** Effect of dinucleotide mismatches on *Pr*Ago. The reaction was performed at 37 °C. The error bars above represent the SDs of three independent experiments

The canonical cleavage mechanism of pAgos relies on the formation of stable DNA duplexes between guides and targets. Therefore, the presence of guide-target mismatches significantly impacts *Pr*Ago’s specific cleavage (Kuzmenko et al. 2019; Liu et al. 2021b). We first analyzed the effects of single mismatches between 5′P guides and target strands on *Pr*Ago activity. As a result, we observed a dramatic reduction in target cleavage efficiency when single mismatches occurred at positions 13 and 15 (Fig. 4A, C). Since the introduction of an additional mismatched nucleotide in the gDNA at a specific position may create a ‘bubble’ in the gDNA-target pairing and impair recognition and cleavage, which benefits SNP detections, we introduced consecutive dinucleotide mismatches at positions 1–18 of the gDNA to analyze the tolerance for guide mismatch (Liu et al. 2021a, b). We discovered that introducing dinucleotide mismatches at positions 4–5 and 6–7 of gDNA could reduce the cleavage efficiency of *Pr*Ago by 20%, while dinucleotide mismatches at positions 5–6 resulted in a 50% decrease in cleavage efficiency. At positions including 10–11, 11–12, 12–13, 13–14, 14–15 and 15–16, *Pr*Ago lost up to 80% of the cleavage activity, and this implied that *Pr*Ago had application prospects in SNP detection (Fig. 4B, D) (Liu et al. 2021a).

### MD simulation analysis for varying target cleavage efficiencies of *Pr*Ago

**Fig. 5:**
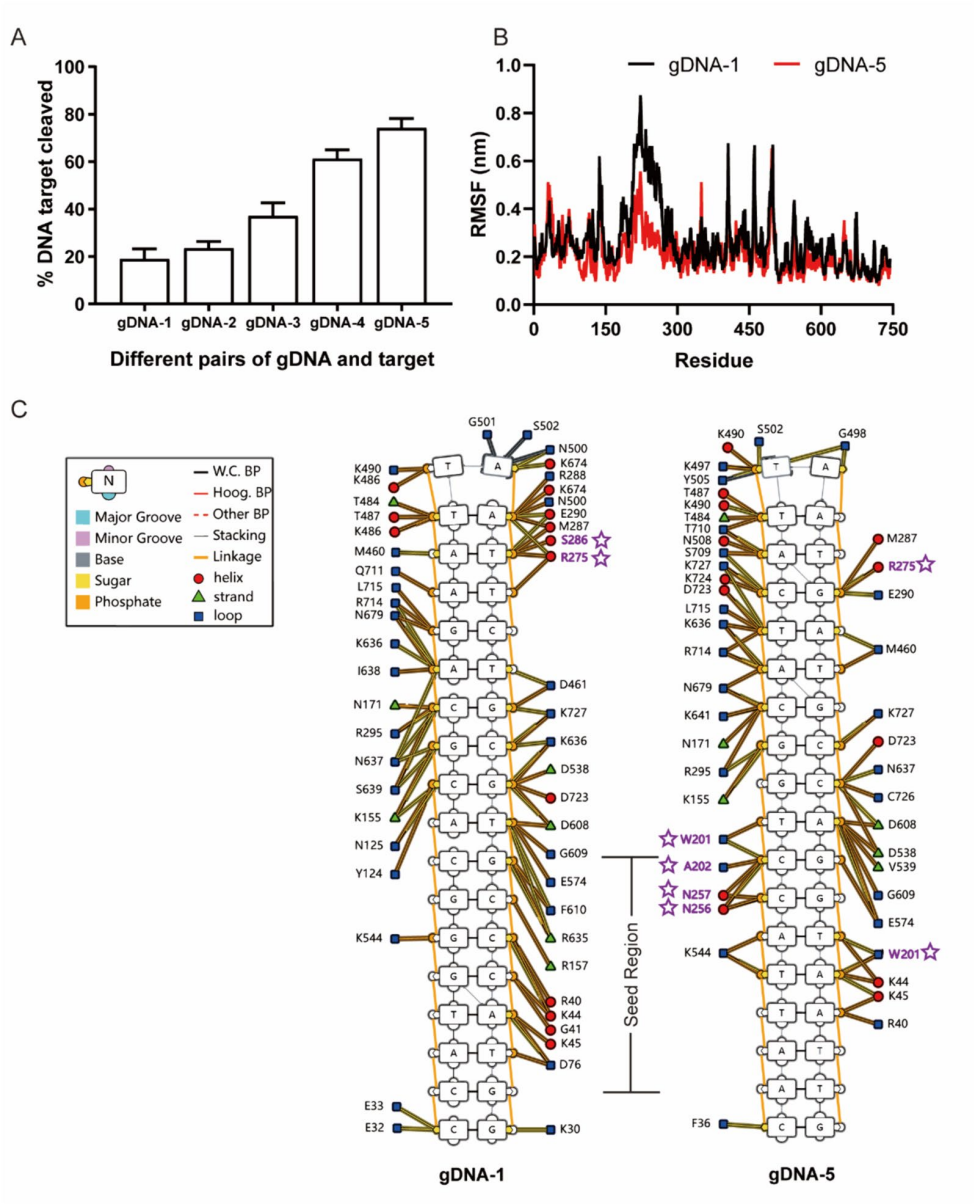
Molecular dynamics simulation analysis of binding differences between *Pr*Ago and gDNA-1/5. **A** Cleavage activity assays of *Pr*Ago with different gDNAs and targets at 37 °C. The experiment was conducted under the condition of pH7.5. The error bars above represent the SDs of three independent experiments. **B** RMSF analysis for gDNA-1/5-*Pr*Ago complexes. **C** The Residue Contact Map shows individual nucleotide-residue interactions, DNA secondary structure, protein secondary structure and DNA interaction moieties. DNA is displayed as a graph, with nucleotides being nodes and edges between them indicating backbone links, base pairing or base stacking. Different base pairing geometries are indicated via the base-pair edges, and other structural features such as backbone breaks, missing phosphates, and DNA strand sense are represented. Protein residues are displayed as small nodes with the node shape and color representing residue secondary structure. Edges between residues and nucleotide nodes represent an interaction between the two and which DNA moiety(s) the interaction involves

Based on the above results, we observed that the specific recognition between the enzyme and the guide-target complex could impact the cleavage efficiency. Therefore, we further focused on the dissection of their interactions at residue level of *Pr*Ago through dynamic simulation. We selected five gDNA sequences similar to those previously reported by Benjamin Ober-Reynolds (Ober-Reynolds et al. 2022). These gDNAs had different sequences matching the target, while the rest remained the same. Although different gDNA sequences perfectly match the target, the cleavage efficiency still varies. Additionally, when the GC content was higher (55%) in three gDNA sequences, the cleavage efficiency was lower. In contrast, the cleavage efficiency was higher in two gDNA sequences with lower GC content (38%) (Fig. 5A, S10A) (Ober-Reynolds et al. 2022).

To analyze the binding affinity between *Pr*Ago and gDNA-1 or 5, we conducted a fluorescence polarization assay to measure the equilibrium dissociation constant (K*D*) using gDNAs labeled with 3′ FAM. Our findings indicated that gDNA-5 demonstrated a higher binding affinity to *Pr*Ago compared to gDNA-1 (Figure S10B) (Huang et al. 2022; Wan et al. 2020; Miyoshi et al. 2016). To gain deeper insights into the potential factors contributing to the differing cleavage efficiencies, we selected gDNA/target-1 and gDNA/target-5 for MD analysis. First, we modeled the 18 nt gDNA and its complementary 18 nt target DNA using Amber software (Heitz and Van Mau 2002). Then, we conducted dsDNA/*Pr*Ago docking using the hDOCK website (Yan et al. 2017). The root mean square deviation (RMSD) from MD simulations was used to describe the conformation movement process of the complex. A larger RMSD value indicated more severe fluctuations and more intense movement, while a stable complex exhibited smaller fluctuations. The RMSD of both systems gradually converged within the first 5 ns of the simulation and maintained stable fluctuations in subsequent simulations (Figure S10C). This suggested that the motion of both complexes stabilizes after kinetic association. Interestingly, the gDNA/target-5-*Pr*Ago complex (red line) had a lower RMSD, indicating greater stability compared to the gDNA/target-1-*Pr*Ago complex. The root mean square fluctuation (RMSF) of each amino acid residue in the system was calculated to further describe structural fluctuations in the system (Fig. 5B). RMSF values of amino acid residues in the system may reflect the relative flexibility and stability of different regions in the receptor during dynamic simulations. After combining gDNA/target-1 with *Pr*Ago, residues near the PAZ domain exhibited greater flexibility than the gDNA/target-5-*Pr*Ago complex. Song et al. mentioned that the PAZ domain was related to loading guide, and the rotation of the PAZ released the guide before pAgo cleavage the target (Song et al. 2004). This increased flexibility in the PAZ domain may contribute to the lower cleavage efficiency of gDNA-1.

To further explore the relationship between the PAZ domain and double stranded nucleic acid, we selected representative moments from previous dynamic simulations. We use DNAproDB (https://dnaprodb.usc.edu) to predict nucleotide–residue contact map interface (Fig. 5C) (Sagendorf et al. 2017, 2020). It was evident that the seed region (positions 2 to 8) of gDNA/target-5 had more *Pr*Ago-bound residues than gDNA/target-1.

## Discussions

Our experiments have confirmed that *Pr*Ago performed the high activity at wide pH range, especially at alkaline pH. Some structural analysis on M-proteases and alkaline cellulases K with different pH-optimum concluded that alkaline adaptation is accompanied by a decreased number of negatively charged amino acids and lysine residues and an increased number of arginine and neutral hydrophilic residues (histidine, asparagine and glutamine) (Shirai et al. 1997, 2001; Vivek et al. 2022). Electrostatic interactions of the charged residues were suggested as a key factor of adaptation to extreme pH. Consequently, substitution of basic by acidic residues was used to improve the charge balance and stability at low pH, and vice versa (Yang et al. 2013). The activity of *Pr*Ago in an alkaline buffer may be attributed to some reasons, unfortunately, we did not obtain the crystal structure, and some speculations require more theoretical support.

*Pr*Ago can cleave DNA when guided by gDNA ranging from 14 to 30 nucleotides in length. We also observed that *Pr*Ago exhibits a remarkable tolerance to single-base mismatches, much like most prokaryotic Agos (Dong et al. 2021). Possibly, most of these mismatches seemed to lead to minor perturbations or weak structural changes within *Pr*Ago, without affecting the formation of stable complexes between *Pr*Ago and mismatched gDNA/target pairs. However, it was notably observed that single-base mismatches at positions 13 or 15 lead to a significant reduction in cleavage efficiency. Therefore, single-base mismatches at positions 13 or 15 hold particular significance, making *Pr*Ago’s ability to discriminate single-base differences a valuable asset for nucleic acid testing applications. When continuous double-base mismatches were introduced at positions such as 5–6, 10–11, 11–12, 12–13, 13–14, 14–15 and 15–16, cleavage efficiency decreased rapidly. It suggested that the cleavage efficiency of enzymes significantly decreased across specific continuous double-base mismatch regions at position 10–16. *Pr*Ago’s ability to distinguished between double-base mismatches could open doors for applications such as gene mutation detection (Liu et al. 2021a).

*Pr*Ago showed significant variations in cleavage efficiency for different guide sequences. Interestingly, when *Pr*Ago interacts with gDNA/target-1, we observed greater instability near the PAZ domain compared to its interaction with gDNA/target-5. The PAZ domain was associated with binding to the 3′ end of the guiding molecule and undergoes rotational changes that facilitated target cleavage by the PIWI domain. We suggested that the reduced cleavage efficiency may be related to the weak binding of gDNA to the PAZ domain (Zander et al. 2014). There were many factors that influenced the effectiveness of targeted cleavage. Further experiments, such as crystal structure analyses of the enzyme–substrate complexes, are necessary to provide valid evidence.

## Conclusions

Given the diverse functions and mechanistic variations of pAgos, it is plausible that many unexplored proteins hold untapped potential, which could address the limitations of currently characterized pAgos. Our study revealed that *Pr*Ago, a multi-turnover enzyme, can cleave ssDNA using 5′-phosphate gDNA, requiring the participation of Mn^2+^ and Mg^2+^ ions. *Pr*Ago can cleave ssDNA across a broad pH range (pH 4–12), with optimal activity at pH 11. As a mesophilic enzyme, it functions at temperatures between 25 and 65 °C, showing peak activity at 65 °C. Furthermore, *Pr*Ago has high tolerance for single-base mismatches, except at positions 13 and 15 of gDNA. Continuous double-base mismatches at positions 10–16 of gDNA significantly reduce cleavage activity. These regions may be targeted in future SNP detection applications involving *Pr*Ago. Additionally, *Pr*Ago mediates DNA-guided DNA cleavage of AT-rich double stranded DNA at 65 °C. Molecular dynamic simulations suggest interactions between the PAZ domain and different nucleic acids strongly influence cleavage efficiency. These results herald a bright future for biotechnological applications in molecular diagnosis and medicine.

## Supplementary Information

Supplementary Material 1: Table S1. Nucleic acids used in this study. Figure S1. Expression and purification of *Pr*Ago protein. Figure S2. The three-dimensional structure model of *Pr*Ago. Figure S3. Comparison of the enzyme cleavage efficiency between *Cb*Ago and *Pr*Ago. Figure S4. The effect of pre-incubating gDNA with *Pr*Ago at different time points on cleavage outcomes. Figure S5. Time gradient of DNA cleavage by *Pr*Ago at high temperatures. Figure S6. Time gradient of DNA cleavage by *Pr*Ago at 300 Mm MnCl_2_. Figure S7. Effect of NaCl concentrations on *Pr*Ago activity mediated by 5′P gDNA. Figure S8. *Pr*Ago is a multi-turnover enzyme-turnover enzyme at 65 °C. Figure S9. Cleavage of pUC19 by *Pr*Ago. Figure S10. Molecular dynamics simulation analysis of binding differences between *Pr*Ago and gDNA-1/5.

## Data Availability

The datasets used and/or analysed during the current study are available from the corresponding author on reasonable request.

## Abbreviations

Ago: Argonaute
pAgo: Prokaryotic Argonaute
*Pr*Ago: *Pseudobutyrivibrio ruminis* Ago
*Ng*Ago: *Natronobacterium gregory* Argonaute
*Cb*Ago: *Clostridium butyricum* Argonaute
ssDNA: Single-strand DNA
dsDNA: Double-strand DNA
MD simulations: Molecular dynamic simulations
*Tt*Ago: *Thermus thermophilus* Ago
5′-P: 5′-Phosphorylated
5′-OH: 5′-Hydroxylated
gDNA: SsDNA guides
gRNA: SsRNA guides
RMSD: Root mean square variance
RMSF: Root mean square fluctuation
*Bl*Ago: *Brevibacillus laterosporus* Ago
*Pb*Ago: *Paenibacillus borealis* Ago
*Km*Ago: *Kurthia massiliensis* Ago
*Rsu*Ago: *Rummeliibacillus suwonensis* Ago
*Mp*Ago: *Marinitoga piezophila* Ago
*Se*Ago: *Synechococcus elongatus* Ago
*Rs*Ago: *Rhodobacter sphaeroides* Ago
hAgo2: *Homo sapiens* Ago
*Lr*Ago: *Limnothrix rosea* Ago
*Kp*Ago: *Kluyveromyces polysporus* Ago
