# Supplementary material for: Characterization of argonaute nucleases from mesophilic bacteria Pseudobutyrivibrio ruminis
Source: Bioresour Bioprocess. 2024 Oct 7;11(1):94. doi: 10.1186/s40643-024-00797-x (PMC11458871; doi:10.1186/s40643-024-00797-x)
Supplement: Supplementary file 1 — Supplementary Material 1: Table S1. Nucleic acids used in this study. Figure S1. Expression and purification of PrAgo protein. Figure S2. The three-dimensional structure model of PrAgo. Figure S3. Comparison of the enzyme cleavage efficiency between CbAgo and PrAgo. Figure S4. The effect of pre-incubating gDNA with PrAgo at different time points on cleavage outcomes. Figure S5. Time gradient of DNA cleavage by PrAgo at high temperatures. Figure S6. Time gradient of DNA cleavage by PrAgo at 300 Mm MnCl2. Figure S7. Effect of NaCl concentrations on PrAgo activity mediated by 5′P gDNA. Figure S8. PrAgo is a multi-turnover enzyme-turnover enzyme at 65 °C. Figure S9. Cleavage of pUC19 by PrAgo. Figure S10. Molecular dynamics simulation analysis of binding differences between PrAgo and gDNA-1/5. [file 40643_2024_797_MOESM1_ESM.docx]

**Supplementary** **figures**


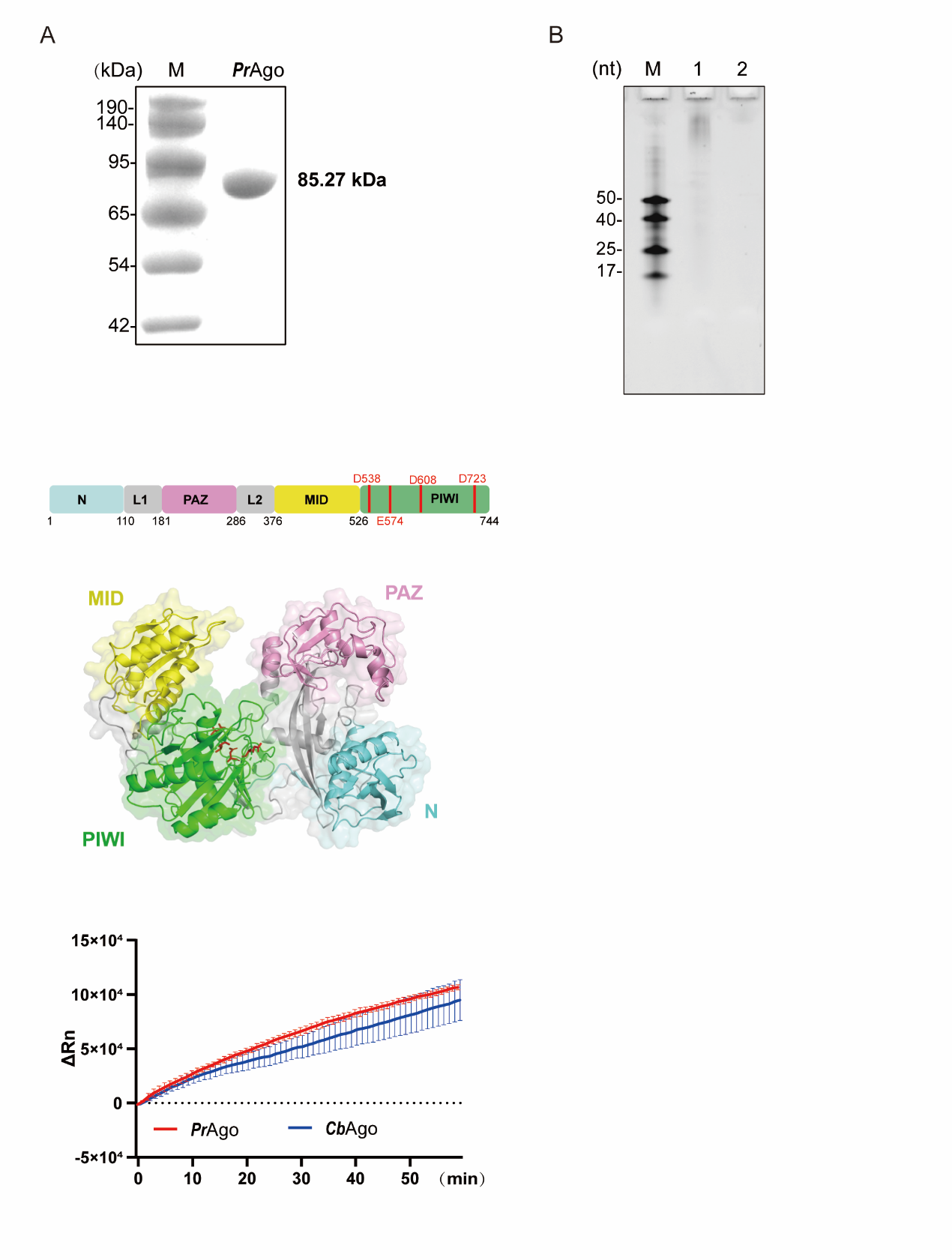


**Figure S1 Expression and purification of *Pr*Ago protein** (A) The purity of *Pr*Ago was determined using SDS-PAGE. (B)Nucleic acids bound to *Pr*Ago with different purification steps. Lane 1 represents the sample after nickel column affinity purification, Lane 2 represents the sample after heparin column affinity purification. After Proteinase K treatment at 58℃ for 2 hours, the remaining nucleic acids were separated on a 16% concentration urea-PAGE gel.


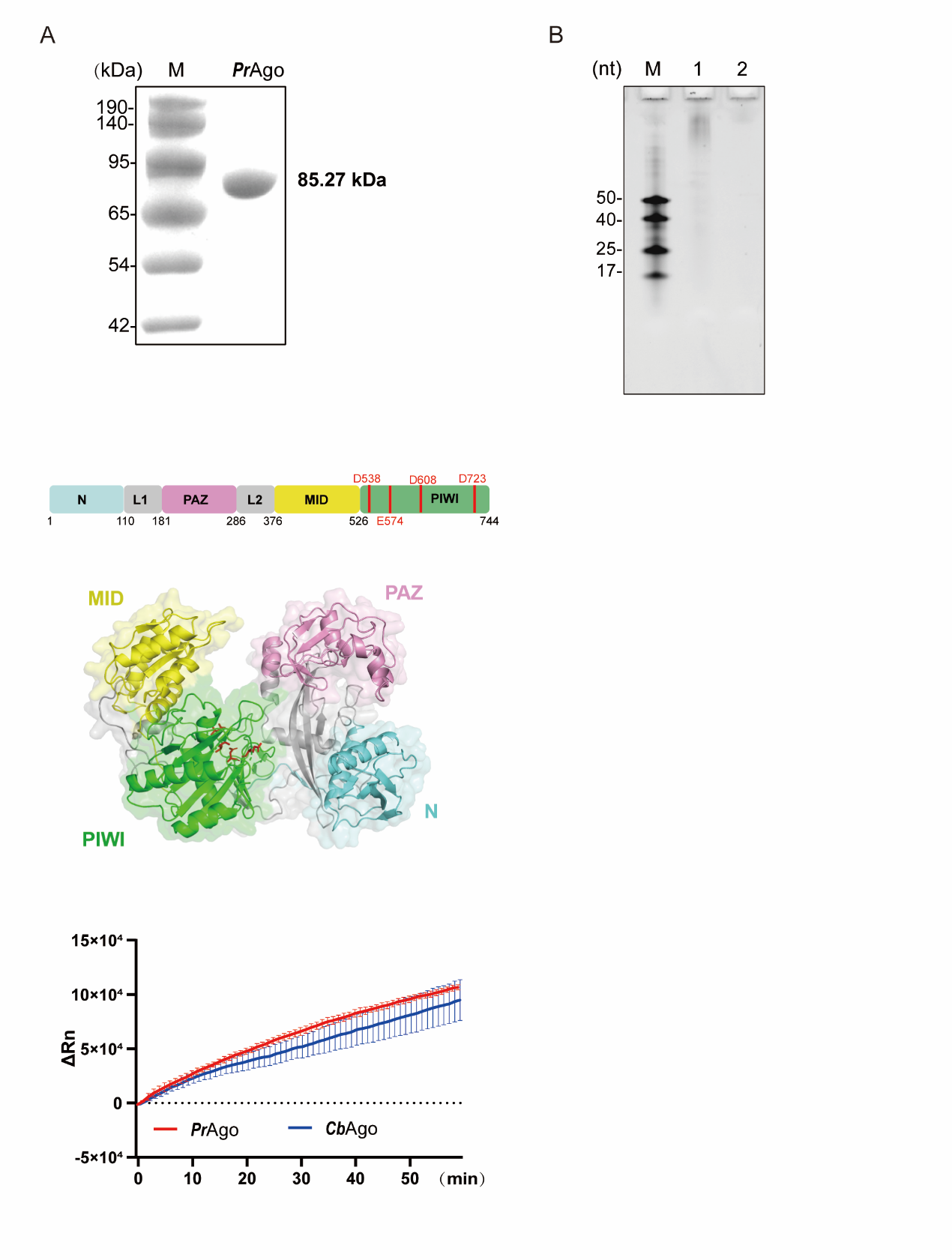


**Figure S2 The three-dimensional structure model of *Pr*Ago** Upper panel: Schematic diagram of the domain organization of *Pr*Ago. L1 and L2 are linker domains. Red mark means the key catalytic residues.Lower panel: Overall 3D structure prediction of the *Pr*Ago domains are coloured according to the colour scheme in panel A.


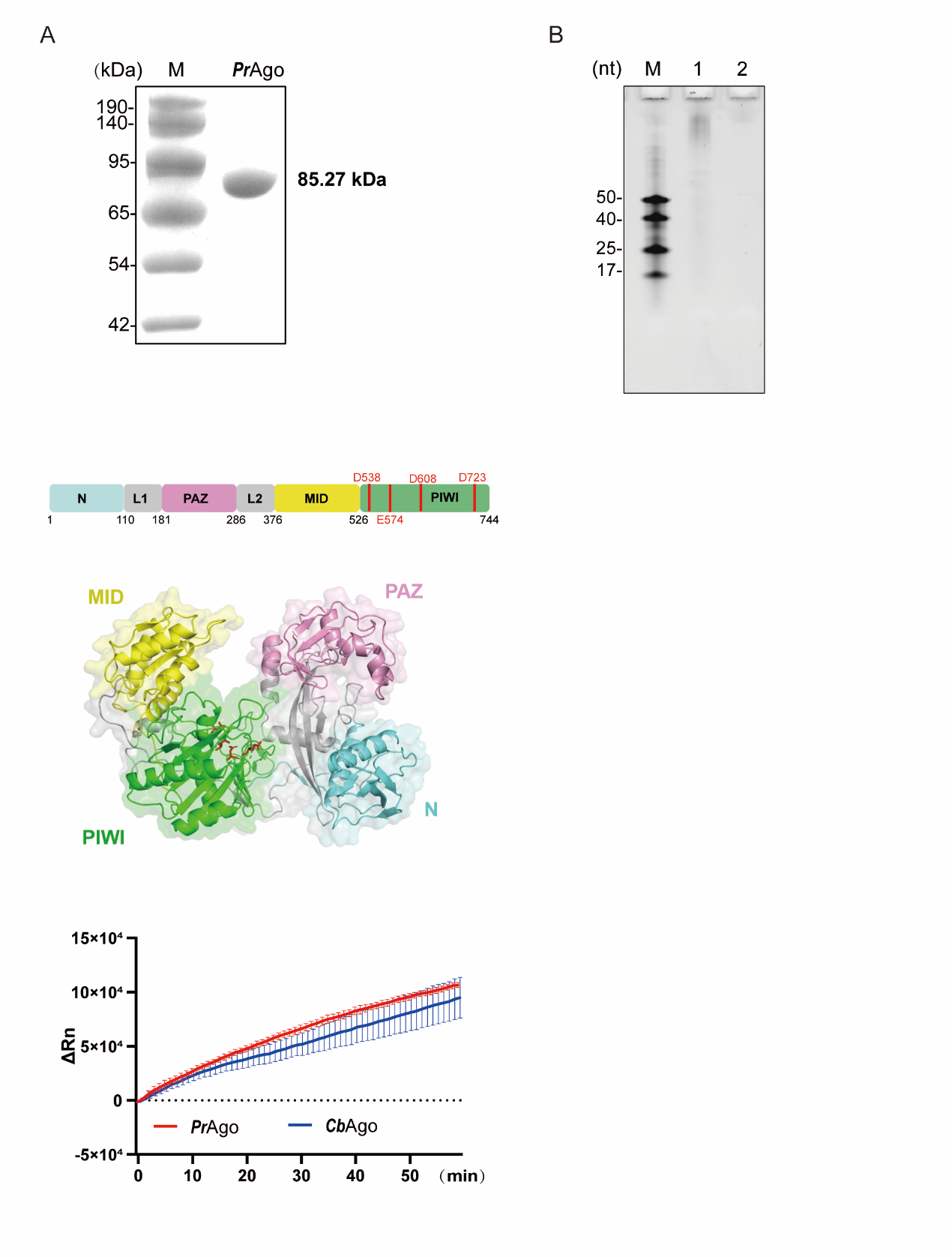


**Figure S3 Comparison of the enzyme cleavage efficiency between *Cb*Ago and *Pr*Ago** 20mM Tris8.0, 3mM MnCl2, 10mM DTT, 0.5M NaCl, 0.5μM gDNA, 0.5μM pAgo，2μM 5' FAM-3'BHQ1-target DNA in reaction buffer. The mixture was observed at 37°C using a qPCR instrument for real-time measuring during 1h. The error bars above represent the SDs of three independent experiments.


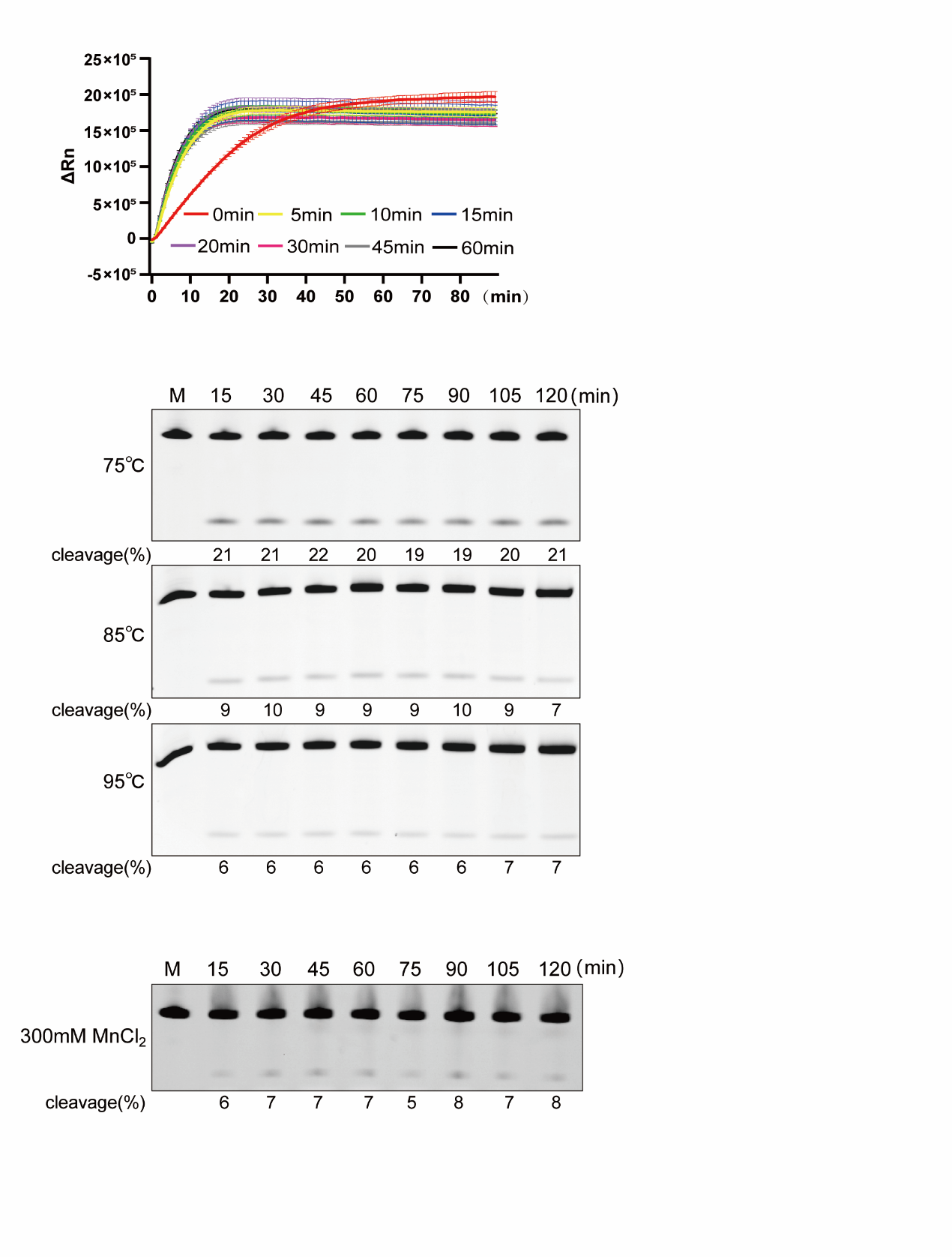


**Figure S4** **The effect of pre-incubating gDNA with *Pr*Ago at different time points on cleavage outcomes** 1uM *Pr*Ago and 1uM gDNA were pre-incubated at room temperature for various duration times, and finally, 2uM 5' FAM-3'BHQ1-target was added. The mixture was observed at 65°C using a qPCR instrument for real-time measuring during 90 minutes. The error bars above represent the SDs of three independent experiments.


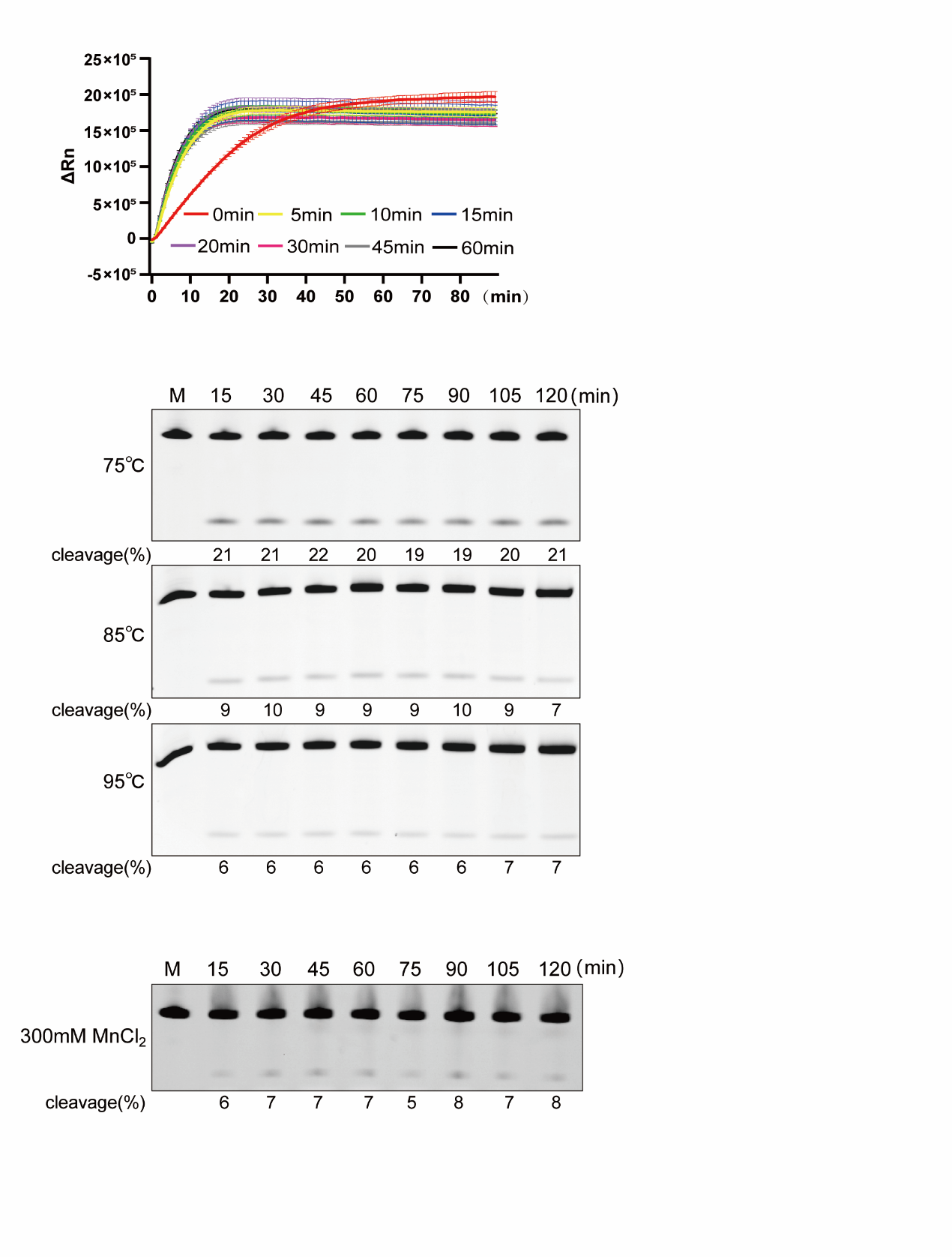


**Figure S5 Time gradient of DNA cleavage by *Pr*Ago at high temperatures.** Reaction buffer contains 20mM Tris 8.0, 1mM NaCl, 3mM MnCl_2_, 10mM DTT, 0.5 μM *Pr*Ago, 0.5 μM gDNA, and 2 μM 5'FAM- target DNA.


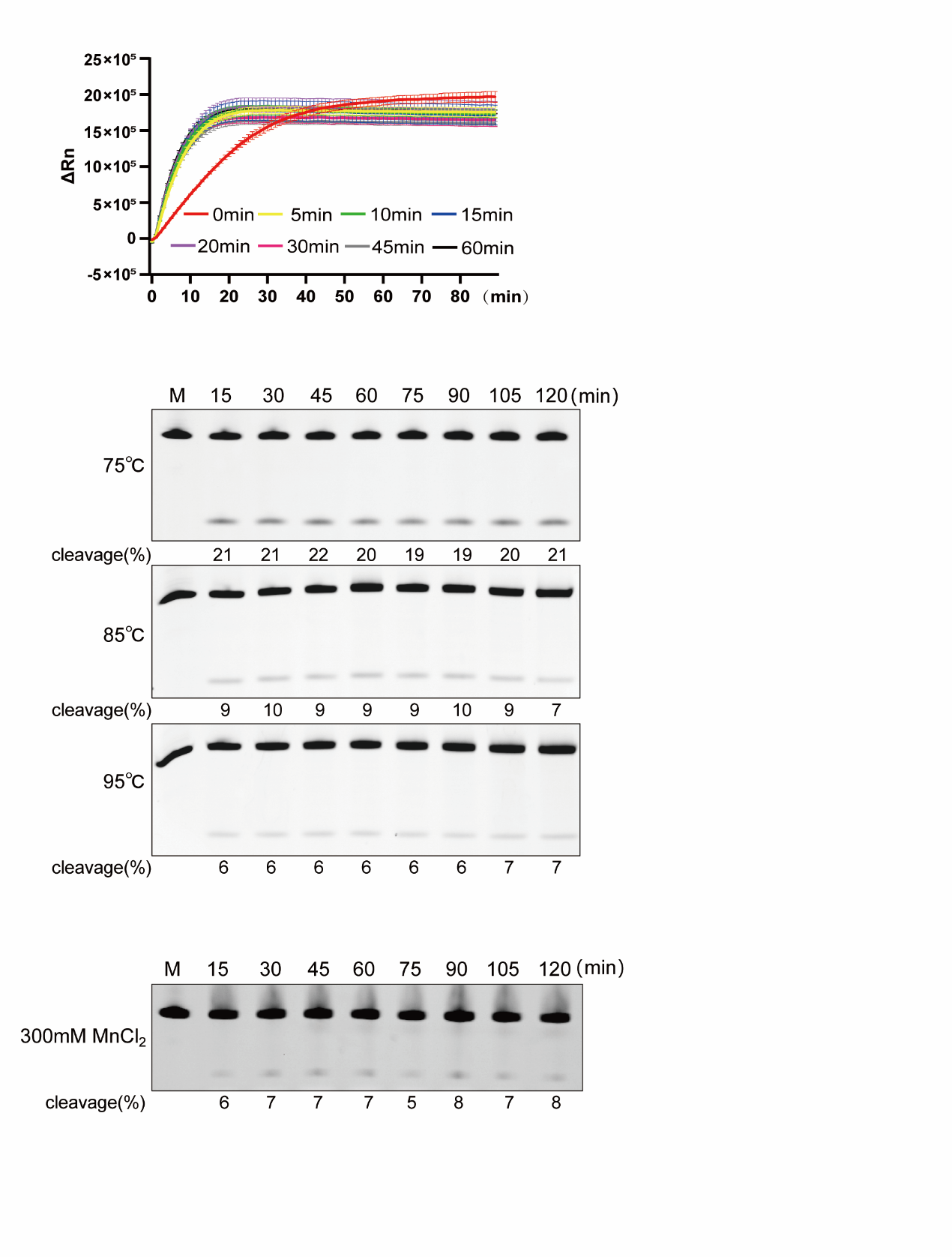


**Figure S6 Time gradient of DNA cleavage by *Pr*Ago at 300Mm MnCl_2_.** Reaction buffer contains 20mM Tris 8.0, 1mM NaCl, 10mM DTT, MnCl_2_, 0.5 μM *Pr*Ago, 0.5 μM gDNA and 2 μM 5'FAM- target DNA.


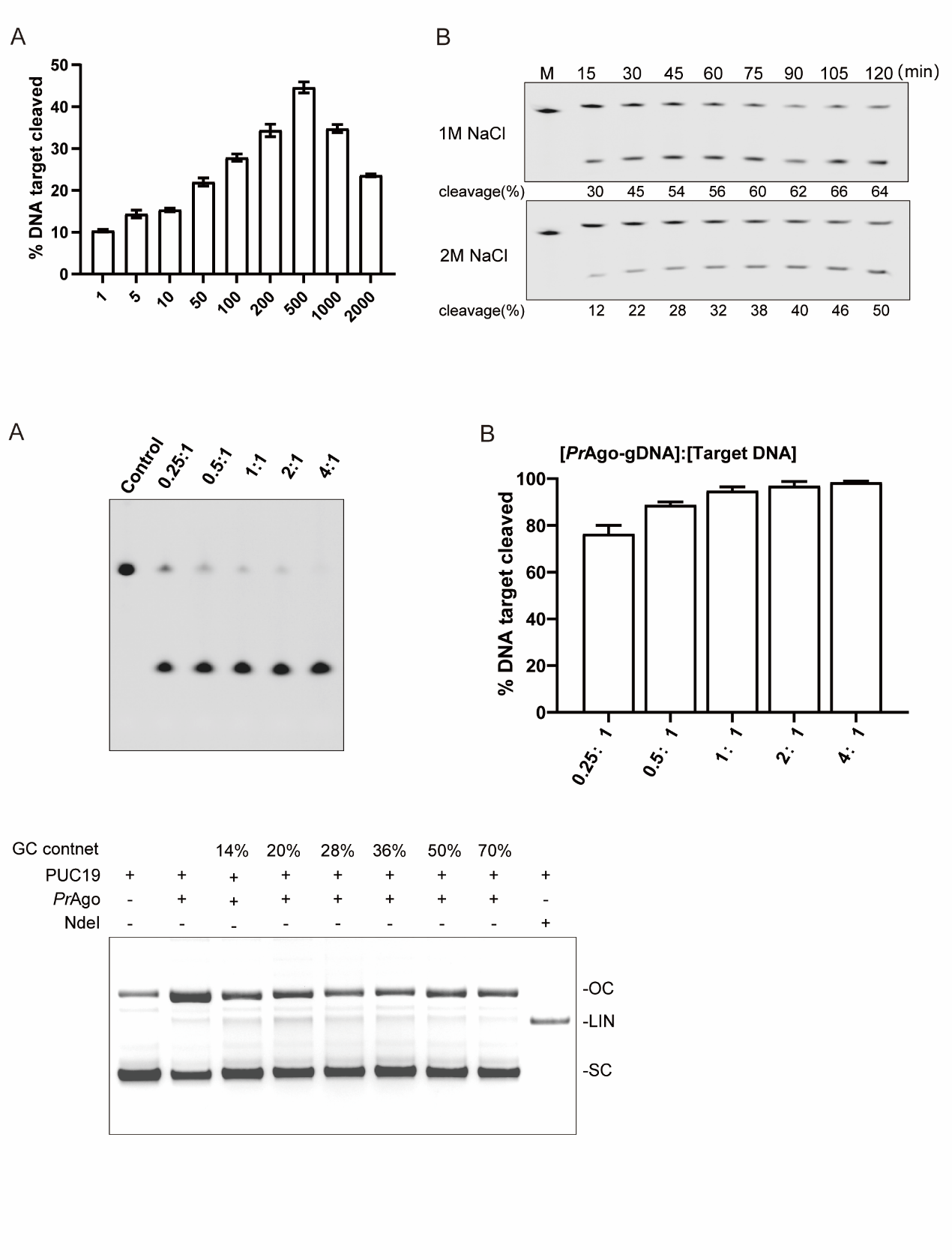


**Figure S7 Effect of NaCl concentrations on *Pr*Ago activity mediated by 5’P gDNA** (A) Reaction buffer contains 20mM Tris 8.0, 3mM MnCl_2_, 10mM DTT, 0.5 μM *Pr*Ago, 0.4 μM gDNA, 2 μM 5'FAM- target DNA and different concentrations of NaCl. Error bars represent the SDs of three independent experiments. (B) Time gradient of DNA cleavage by *Pr*Ago at 1M or 2M NaCl.


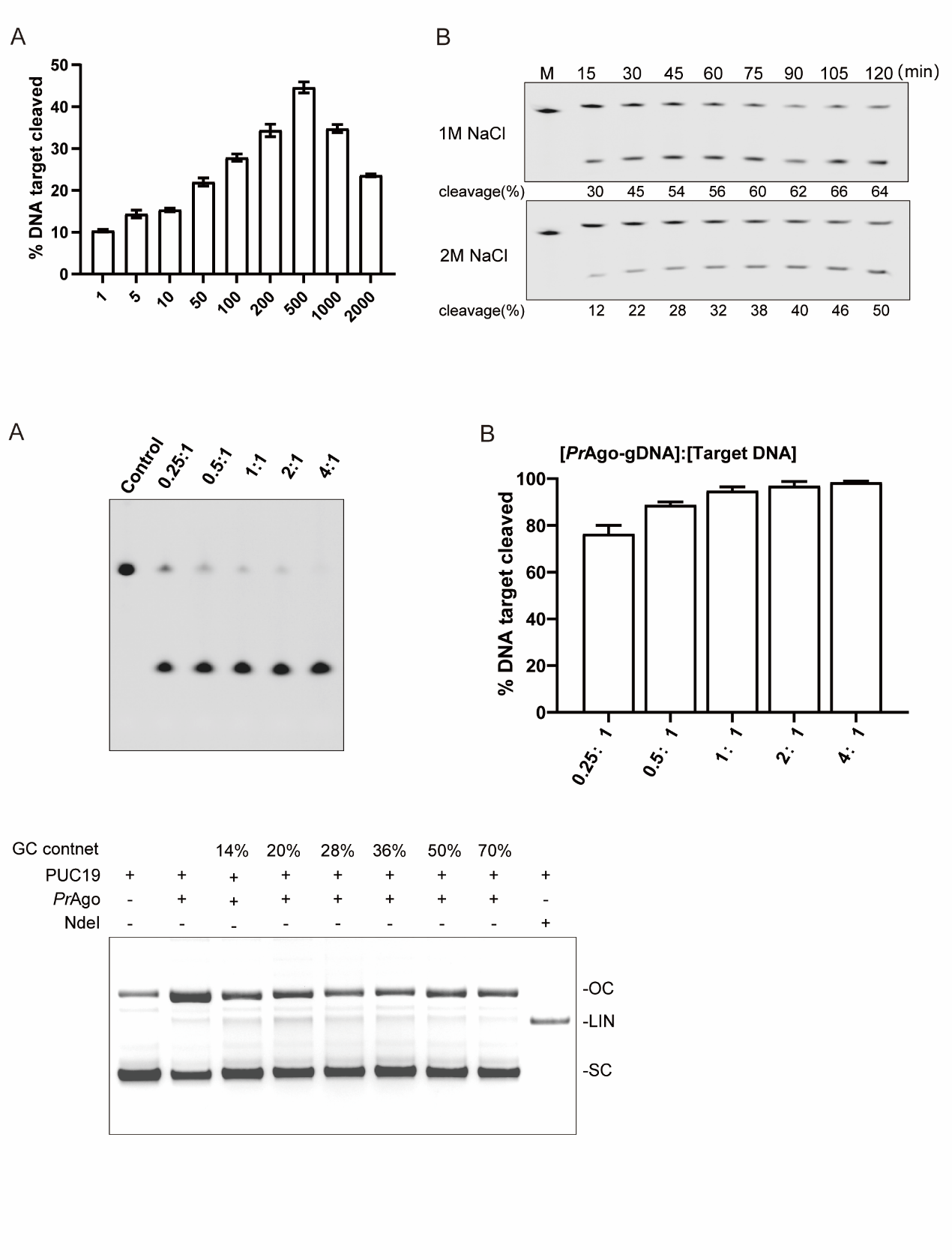


**Figure S8 *Pr*Ago is a multi-turnover enzyme-turnover enzyme at 65°C** (A) When the target DNA is constant, the influence of different ratios of [*Pr*Ago-gDNA]: Target DNA on cleavage was examined. 1 μM of target DNA were added according to the proportions illustrated in the figure. The reaction was conducted at 65°C for 4 hours, and the reaction products were separated on a 16% urea-PAGE gel. (B) Statistical analysis of Figure A was performed. The results from three repeated experiments were compiled and presented as a bar chart. The error bars depicted above represent the standard deviations of three independent experiments.


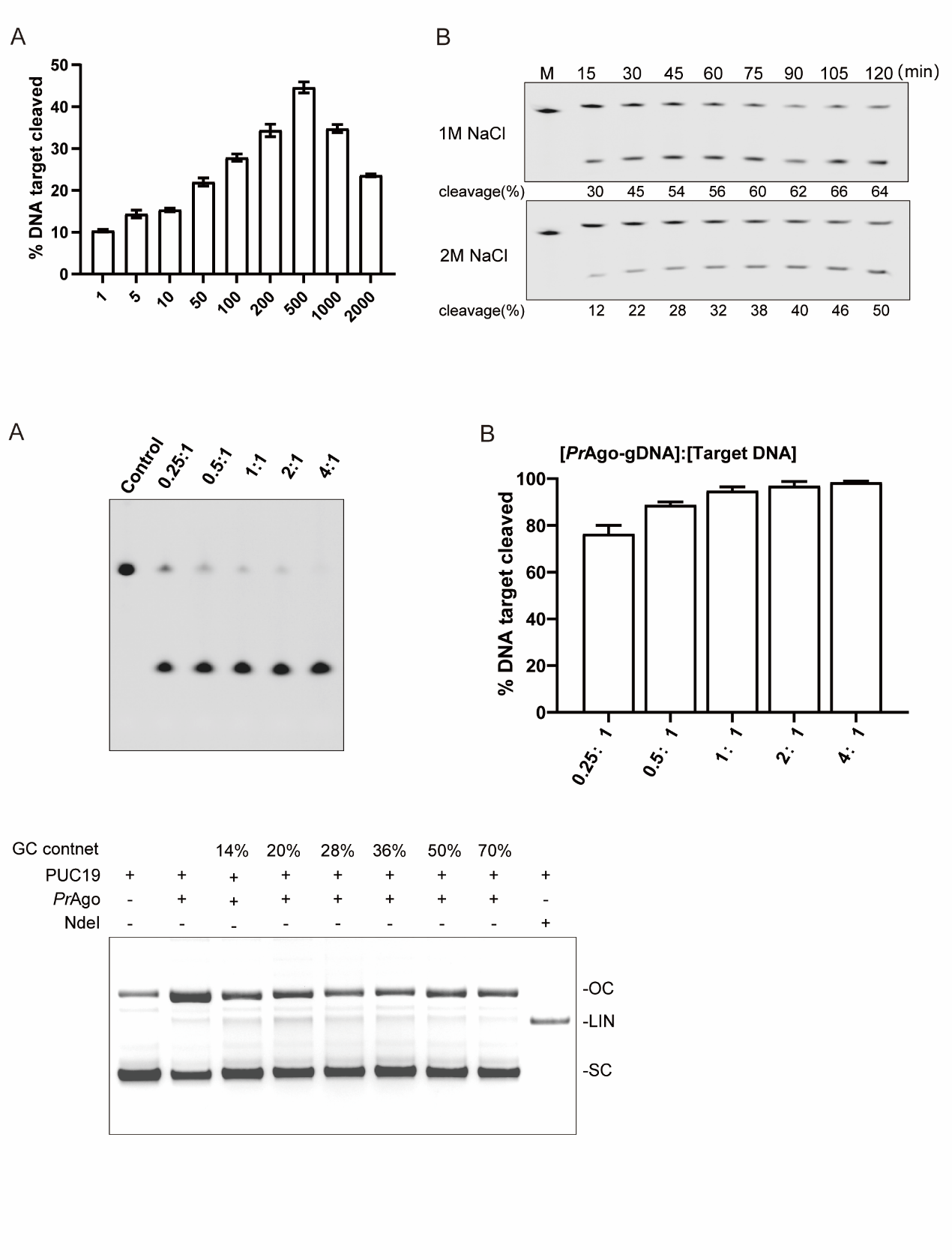


**Figure S9 Cleavage of pUC19 by *Pr*Ago** Plasmid cleavage in different target regions at 37°C for 3h. Reaction buffer contains 20mM Tris 8.0, 1mM NaCl, 3mM MnCl2, 10mM DTT, 0.5 μM PrAgo, 0.5 μM gDNA, 700ng pUC19. OC, open circular plasmid; LIN linearized plasmid; SC, supercoiled plasmid.

**
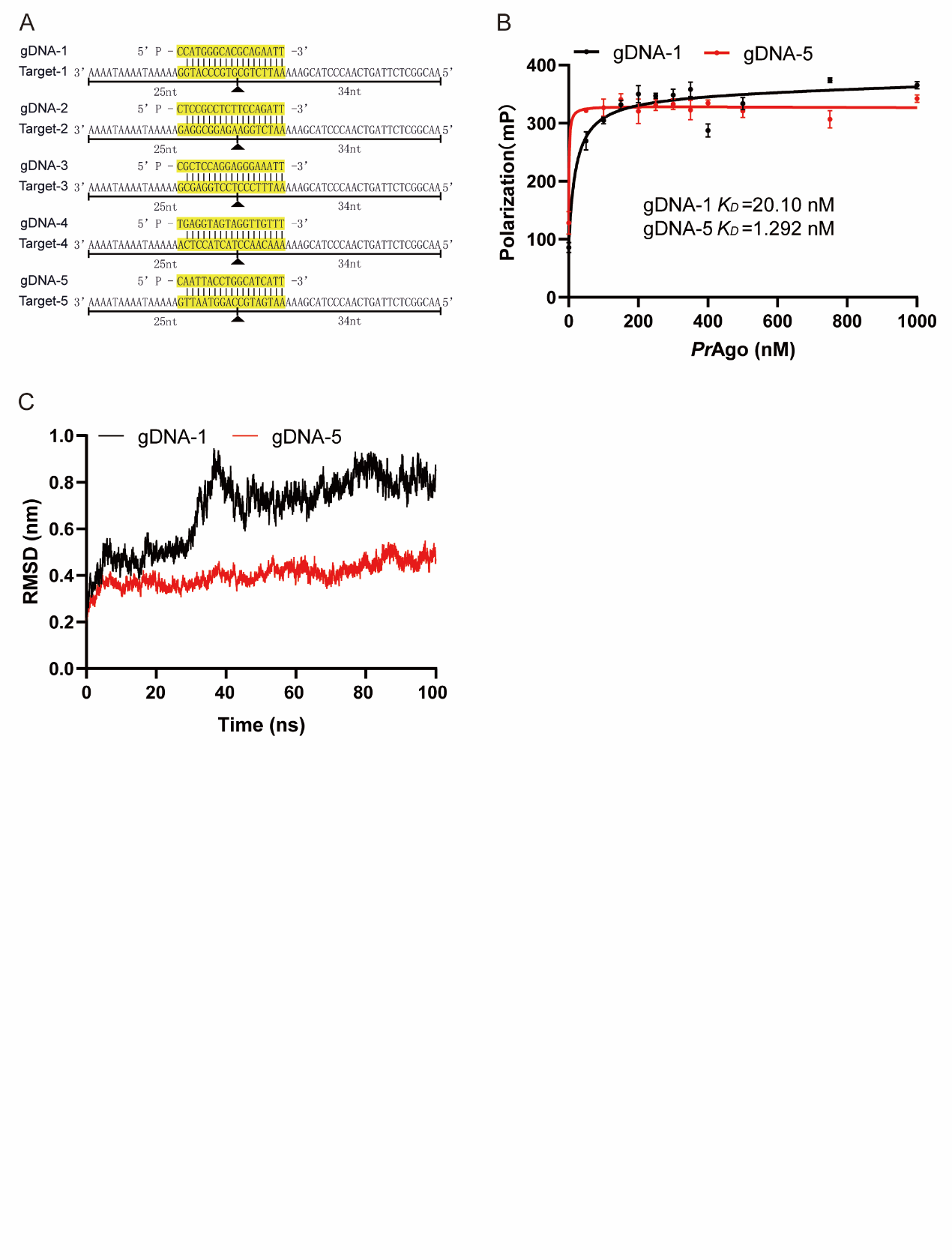
**

**Figure S10 Molecular dynamics simulation analysis of binding differences between *Pr*Ago and gDNA-1/5** (A) Schematic of different gDNAs and targets. Yellow markings indicated different sequences. The cleavage positions were indicated with a black triangle, black lines indicating the predicted 25- and 34-nt cleavage products. (B) Fluorescence polarization detection of the equilibrium dissociation constant (K*D*) of *Pr*Ago for different guides. Incubated 0-1000 nM *Pr*Ago with 5 nM 5'P-3'FAM-gDNA in reaction buffer at 37°C for 15 minutes, and measured the polarization using a Spark microplate reader. Performed nonlinear regression fitting using GraphPad Prism to calculate K*D*. The error bars above represent the SDs of three independent experiments. (C) RMSD analysis for gDNA 1/5 -*Pr*Ago complexes.

**Supplementary tables**

**Table S1** **Nucleic acids used in this study**

| Name | Sequence (5'-3') |
| --- | --- |
| 5'P-gDNA -5(18nt) | P -CAATTACCTGGCATCATT |
| 5'OH-gDNA -5(18nt) | OH -CAATTACCTGGCATCATT |
| 5'P-gRNA -5(18nt) | P -CAAUUACCUGGCAUCAUU |
| 5'OH-gRNA -5(18nt) | OH -CAAUUACCUGGCAUCAUU |
| DNA -target-5(59nt) | AACGGCTCTTAGTCAACCCTACGAAAAATGATGCCAGGTAATTGAAAAATAAAATAAAA |
| RNA -target-5(59nt) | AACGGCUCUUAGUCAACCCUACGAAAAAUGAUGCCAGGUAAUUGAAAAAUAAAAUAAAA |
| 5'P-gDNA -1(18nt) | P -CCATGGGCACGCAGAATT |
| DNA -target-1(86nt) | GGCGACGCATCTAACGGCTCTTAGTCAACCCTACGAAAAATTCTGCGTGCCCATGGAAAAATAAAATAAAATAAAATAAAATAAAA |
| 5'P-gDNA -2(18nt) | P -CTCCGCCTCTTCCAGATT |
| DNA -target-2(86nt) | GGCGACGCATCTAACGGCTCTTAGTCAACCCTACGAAAAATCTGGAAGAGGCGGAGAAAAATAAAATAAAATAAAATAAAATAAAA |
| 5'P-gDNA -3(18nt) | P -CGCTCCAGGAGGGAAATT |
| DNA -target-3(86nt) | GGCGACGCATCTAACGGCTCTTAGTCAACCCTACGAAAAATTTCCCTCCTGGAGCGAAAAATAAAATAAAATAAAATAAAATAAAA |
| 5'P-gDNA -4(18nt) | P -TGAGGTAGTAGGTTGTTT |
| DNA -target-4(86nt) | GGCGACGCATCTAACGGCTCTTAGTCAACCCTACGAAAAAACAACCTACTACCTCAAAAAATAAAATAAAATAAAATAAAATAAAA |
| DNA -target-5(86nt) | GGCGACGCATCTAACGGCTCTTAGTCAACCCTACGAAAAATGATGCCAGGTAATTGAAAAATAAAATAAAATAAAATAAAATAAAA |
| Probe gDNA (16nt) | ACCACCACGATGAAAA |
| Probe (40nt) | 5’FAM-ACATTAGTAAACAACCTACTACCTCATATTGTTAATGCAC-3’BHQ1 |
| 5'P-gDNA -6(35 nt ) | P-CGAGGTAGTAGGTTGTATAGTATATTAAATTATTT |
| 5'P-gDNA -6(30 nt ) | P-CGAGGTAGTAGGTTGTATAGTATATTAAAT |
| 5'P-gDNA -6(25 nt ) | P-CGAGGTAGTAGGTTGTATAGTATAT |
| 5'P-gDNA -6(21 nt ) | P-CGAGGTAGTAGGTTGTATAGT |
| 5'P-gDNA -6(18 nt ) | P-CGAGGTAGTAGGTTGTAT |
| 5'P-gDNA -6(16 nt ) | P-CGAGGTAGTAGGTTGT |
| 5'P-gDNA -6(15 nt ) | P-CGAGGTAGTAGGTTG |
| 5'P-gDNA -6(14 nt) | P-CGAGGTAGTAGGTT |
| 5'P-gDNA -6(13 nt ) | P-CGAGGTAGTAGGT |
| 5'P-gDNA -6(12 nt ) | P-CGAGGTAGTAGG |
| 5'P-gDNA -6(10 nt ) | P-CGAGGTAGTA |
| 5'P-gDNA -6(8 nt ) | P-CGAGGTAG |
| 5'P-gDNA -5-mis-1-A | P -AAATTACCTGGCATCATT |
| 5'P-gDNA -5-mis-1-T | P -TAATTACCTGGCATCATT |
| 5'P-gDNA -5-mis-1 | P -GAATTACCTGGCATCATT |
| 5'P-gDNA -5-mis-2 | P -CTATTACCTGGCATCATT |
| 5'P-gDNA -5-mis-3 | P -CATTTACCTGGCATCATT |
| 5'P-gDNA -5-mis-4 | P -CAAATACCTGGCATCATT |
| 5'P-gDNA -5-mis-5 | P -CAATAACCTGGCATCATT |
| 5'P-gDNA -5-mis-6 | P -CAATTTCCTGGCATCATT |
| 5'P-gDNA -5-mis-7 | P -CAATTAGCTGGCATCATT |
| 5'P-gDNA -5-mis-8 | P -CAATTACGTGGCATCATT |
| 5'P-gDNA -5-mis-9 | P -CAATTACCAGGCATCATT |
| 5'P-gDNA -5-mis-10 | P -CAATTACCTCGCATCATT |
| 5'P-gDNA -5-mis-11 | P -CAATTACCTGCCATCATT |
| 5'P-gDNA -5-mis-12 | P -CAATTACCTGGGATCATT |
| 5'P-gDNA -5-mis-13 | P -CAATTACCTGGCTTCATT |
| 5'P-gDNA -5-mis-14 | P -CAATTACCTGGCAACATT |
| 5'P-gDNA -5-mis-15 | P -CAATTACCTGGCATGATT |
| 5'P-gDNA -5-mis-16 | P -CAATTACCTGGCATCTTT |
| 5'P-gDNA -5-mis-17 | P -CAATTACCTGGCATCAAT |
| 5'P-gDNA -5-mis-18 | P -CAATTACCTGGCATCATA |
| 5'P-gDNA -5-doub-mis-1,2 | P -GTATTACCTGGCATCATT |
| 5'P-gDNA -5-doub-mis-2,3 | P -CTTTTACCTGGCATCATT |
| 5'P-gDNA -5-doub-mis-3,4 | P -CATATACCTGGCATCATT |
| 5'P-gDNA -5-doub-mis-4,5 | P -CAAAAACCTGGCATCATT |
| 5'P-gDNA -5-doub-mis-5,6 | P -CAATATCCTGGCATCATT |
| 5'P-gDNA -5-doub-mis-6,7 | P -CAATTTGCTGGCATCATT |
| 5'P-gDNA -5-doub-mis-7,8 | P -CAATTAGGTGGCATCATT |
| 5'P-gDNA -5-doub-mis-8,9 | P -CAATTACGAGGCATCATT |
| 5'P-gDNA -5-doub-mis-9,10 | P -CAATTACCACGCATCATT |
| 5'P-gDNA -5-doub-mis-10,11 | P -CAATTACCTCCCATCATT |
| 5'P-gDNA -5-doub-mis-11,12 | P -CAATTACCTGCGATCATT |
| 5'P-gDNA -5-doub-mis-12,13 | P -CAATTACCTGGGTTCATT |
| 5'P-gDNA -5-doub-mis-13,14 | P -CAATTACCTGGCTACATT |
| 5'P-gDNA -5-doub-mis-14,15 | P -CAATTACCTGGCAAGATT |
| 5'P-gDNA -5-doub-mis-15,16 | P -CAATTACCTGGCATGTTT |
| 5'P-gDNA -5-doub-mis-16,17 | P -CAATTACCTGGCATCTAT |
| 5'P-gDNA -5-doub-mis-17,18 | P -CAATTACCTGGCATCAAA |
| FW 5'P gDNA (14% GC of target region) | P-TTAGATTGATTTAAAACTTCA |
| RV 5'P gDNA (14% GC of target region) | P-TGAAGTTTTAAATCAATCTAA |
| FW 5'P gDNA (20% GC of target region) | P-TTAAAACTTCATTTTTAATTT |
| RV 5'P gDNA (20% GC of target region) | P-AAATTAAAAATGAAGTTTTAA |
| FW 5'P gDNA (28% GC of target region) | P-TTACTCATATATACTTTAGAT |
| RV 5'P gDNA (28% GC of target region) | P-ATCTAAAGTATATATGAGTAA |
| FW 5'P gDNA (36% GC of target region) | P-TCTCATGACCAAAATCCCTTA |
| RV 5'P gDNA (36% GC of target region) | P-TAAGGGATTTTGGTCATGAGA |
| FW 5'P gDNA (50% GC of target region) | P-CGTGAGTTTTCGTTCCACTGA |
| RV 5'P gDNA (50% GC of target region) | P-TCAGTGGAACGAAAACTCACG |
| FW 5'P gDNA (70% GC of target region) | P-CGCCCTGACGGGCTTGTCTGC |
| RV 5'P gDNA (70% GC of target region) | P-GCAGACAAGCCCGTCAGGGCG |
